# Impact of Stoichiometry Representation on Simulation of Genotype-Phenotype Relationships in Metabolic Networks

**DOI:** 10.1371/journal.pcbi.1002758

**Published:** 2012-11-01

**Authors:** Ana Rita Brochado, Sergej Andrejev, Costas D. Maranas, Kiran R. Patil

**Affiliations:** 1 Structural and Computational Biology Unit, European Molecular Biology Laboratory, Heidelberg, Germany; 2 Center for Microbial Biotechnology, Department of Systems Biology, Technical University of Denmark, Lyngby, Denmark; 3 Department of Chemical Engineering, Pennsylvania State University, University Park, Pennsylvania, United States of America; University of Virginia, United States of America

## Abstract

Genome-scale metabolic networks provide a comprehensive structural framework for modeling genotype-phenotype relationships through flux simulations. The solution space for the metabolic flux state of the cell is typically very large and optimization-based approaches are often necessary for predicting the active metabolic state under specific environmental conditions. The objective function to be used in such optimization algorithms is directly linked with the biological hypothesis underlying the model and therefore it is one of the most relevant parameters for successful modeling. Although linear combination of selected fluxes is widely used for formulating metabolic objective functions, we show that the resulting optimization problem is sensitive towards stoichiometry representation of the metabolic network. This undesirable sensitivity leads to different simulation results when using numerically different but biochemically equivalent stoichiometry representations and thereby makes biological interpretation intrinsically subjective and ambiguous. We hereby propose a new method, Minimization of Metabolites Balance (MiMBl), which decouples the artifacts of stoichiometry representation from the formulation of the desired objective functions, by casting objective functions using metabolite turnovers rather than fluxes. By simulating perturbed metabolic networks, we demonstrate that the use of stoichiometry representation independent algorithms is fundamental for unambiguously linking modeling results with biological interpretation. For example, MiMBl allowed us to expand the scope of metabolic modeling in elucidating the mechanistic basis of several genetic interactions in *Saccharomyces cerevisiae*.

## Author Summary

One of the challenging tasks in systems biology is to quantitatively predict the metabolic behavior of the cell under given genetic and environmental constraints. To this end, genome-scale metabolic reconstructions and simulation tools are indispensable. The choice of the objective function to be used for simulating genome-scale metabolic models is dependent on the biological context and one of the most relevant parameters for successful modeling. Formulation of the intended objective function often requires the use of multiple fluxes, e.g. the sum of fluxes through ATP-producing reactions. We demonstrate that the existing tools confound biological interpretation of the simulations due to undesired dependence on the representation of stoichiometry and propose a new tool – Minimization of Metabolites Balance (MiMBl). MiMBl allows casting of the desired biological objective functions into linear optimization models and gives consistent simulation results when using numerically different but biochemically equivalent stoichiometry representations. We demonstrate relevance of MiMBl for addressing biological questions through improved predictions of genetic interactions within the yeast metabolic network. Genetic interactions imply functional relationship between the genes and therefore allow assessing different hypotheses for the underlying biological principles. MiMBl explains several of the genetic interactions as outcome of flux re-routing for minimal metabolite turnover adjustments.

## Introduction

The fundamental role of metabolism within a living cell has become a focal point of study in many disciplines, such as cell biology, physiology, medicine and synthetic biology. The assembly of all reactions and metabolites into a genome-scale metabolic network provides a comprehensive structural framework for integrative data analysis [1], [2], as well as for quantitative modeling of cellular metabolism [3]–[6]. As the solution space for the metabolic flux state of the cell is typically very large, constraint based optimization approaches are often applied for simulating metabolic fluxes. In essence, these approaches search for an optimal flux distribution that maximizes or minimizes an appropriate biological objective function while satisfying the mass balance and metabolite exchange constraints. Among these, Flux Balance Analysis [7] is a widely used simulation tool that utilizes a linear programming formulation for maximization of growth (synthesis of biomass constituents) as biological objective function. FBA has been applied with various degrees of success, albeit mostly for “wild-type” or unperturbed metabolic networks [8], [9]. In addition to FBA, various other objective functions are frequently used, including minimization of overall intracellular flux and maximization of ATP yield, among others. An overview of various commonly used objective functions and their evaluation against experimental data for *Escherichia coli* can be found in Schuetz *et al.* [10]. In case of genetically or environmentally perturbed networks, Minimization of Metabolic Adjustment algorithm - MoMA [11] - has been reported to better represent the biological observations [11]–[14]. The hypothesis underlying MoMA is that fluxes in a perturbed cell (*e.g.* a mutant) will be redistributed so as to be as similar as possible to the wild-type.

The biological principles exemplified by simulation tools for both wild-type and perturbed networks are undeniably fascinating, which is confirmed by their numerous applications – including prediction of genetic interactions [2], [15], [16], metabolic engineering [13], [14], [17], microbial community modeling [18], [19] and search for evolutionary constraints in relation to different objective functions [20]. Several of the objective functions commonly used in these and other applications rely on the use of linear combination of fluxes, e.g., MoMA or minimization of overall intracellular flux (**Table 1**). We found that the mathematical formulation of this class of problems (*i.e.* where linear combination of fluxes is part of the objective function) is sensitive to the representation of the reaction stoichiometry, with results strongly dependent on the adopted scaling of the stoichiometric coefficients. Such dependency confounds the biological interpretation of simulation results, as biochemically equivalent alternative representations of the same network can lead to contradictory predictions upon a given genetic or environmental perturbation. For example, the status of a given gene may change from non-essential to essential while using biochemically equivalent representations of the stoichiometry of the metabolic network (**Table S1**). As the stoichiometric representation of any reaction is subjective (often scaled to have coefficient of 1 for one of the reactants/products) and a typical genome-scale modeling problem involves hundreds of reactions, there are infinitely many biochemically equivalent ways to represent a given metabolic network. Any simulation algorithm should therefore be independent of the stoichiometry representation.

**Table 1:** Formulation of different biological objective functions using MiMBl.

Biological objective function	Previous objective function	Description	MiMBl objective function	Description
Minimization of metabolic adjustment	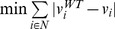 [11]	Minimization of Manhattan distance between the vectors containing the reference and mutant flux distributions	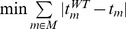	Minimization of Manhattan distance between the vectors containing the reference and mutant intracellular metabolites turnover
Minimization of overall intracellular flux	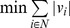 [23]	Minimization of the sum of all intracellular fluxes	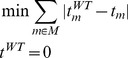	Minimization of the sum of intracellular metabolites turnover
Maximization or Minimization of ATP yield	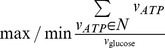 [35], [36]	Max/Minimization of the sum of all reactions producing ATP	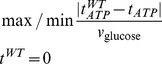	Max/Minimization of ATP turnover
Minimization of redox potential	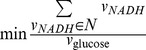 [35]	Minimization of the sum of all reactions producing NADH	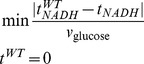	Minimization of NADH turnover
Maximization of biomass*	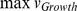 [7], [37]	Maximization of biomass flux	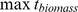	Maximization of biomass turnover

* **Note:** Biomass production within metabolic models is typically represented as a single reaction accounting for all the biomass constitutes. Therefore, FBA and MiMBl are equivalent for maximizing biomass.

We motivate the need for rethinking the problem formulation for metabolic modeling by illustrating how the current methods lead to incoherent biological predictions when alternatively representing the reaction stoichiometry. Tackling a proper problem formulation, we propose a new methodology for metabolic modeling – **Mi**nimization of **M**etabolites **B**a**l**ance (MiMBl), which accounts for reaction stoichiometry in the objective function by mapping the flux space into the metabolite turnover space. As intended, MiMBl shows robust predictions independently of the stoichiometry representation. We demonstrate the biological relevance of the new formulation with increased power for predicting genetic interactions in the metabolic network of *S. cerevisiae*. In a recent study reporting a large genetic interactions dataset covering the *S. cerevisiae* metabolic network [2], FBA was found to have limited capability for predicting the experimentally observed interactions, partially due to the lack of regulatory information. Within this study we successfully challenged MiMBl to accomplish the task of extending the range of genetic interactions that can be predicted. By combining the results from MiMBl and FBA, we probe the operating mechanisms underlying genetic interactions within metabolic networks.

## Results/Discussion

Several of the biological objective functions widely used in metabolic modeling are currently formulated as linear (or quadratic) combination of fluxes. Minimization of sum of intracellular fluxes and minimization of metabolic adjustment belong to this class and are herein used as case studies of biological principles that can be robustly formulated by using MiMBl. Two different genome-scale reconstructions of the *S. cerevisiae* metabolic network are used, viz. *i*FF708 [21] and *i*AZ900 [22], as the choice of the appropriate metabolic reconstruction depends on the biological question to be addressed (**Methods**).

### Stoichiometry representation and minimization of sum of fluxes

**Figure 1:**
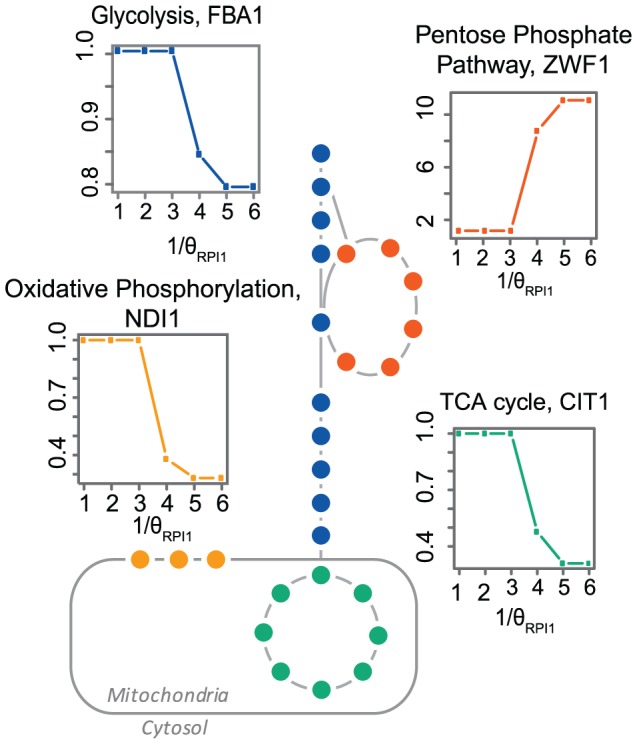
Minimization of overall intracellular flux leads to divergent predictions for flux distribution when using biochemically equivalent stoichiometry representations. Shown are predicted fluxes through key pathways within the *S. cerevisiae* central carbon metabolism, using numerically different but biochemically equivalent stoichiometric representation of reaction *RPI1* (θ*_RPI1_*, **Methods**). θ*_RPI1_* is represented on the x-axis, while fold-change of fluxes relatively to θ = 1 is represented on the y-axis. A representative reaction from each of the pathways was selected to illustrate the flux re-arrangement; *FBA1* for glycolysis, *ZWF1* for pentose phosphate pathway, *CIT1* for tricarboxilic acid cycle and *NID1* for oxidative phosphorylation. Note that θ = 1 is an arbitrary reference, as the stoichiometric representation of any reaction is subjective, often scaled to have coefficient of 1 for one of the reactants/products.

Minimization of the sum of intracellular flux is a routinely used objective function for estimating intracellular fluxes [10], [20], [23], [24]. By using the *i*FF708 *S. cerevisiae* genome-scale metabolic reconstruction [21] together with experimentally determined exchange rate constraints (**Text S1**), we illustrate how the use of this objective function leads to inconsistent predictions when using numerically different, but biochemically equivalent, reaction stoichiometry. Linear scaling of all stoichiometric coefficients of a given reaction (*e.g.* multiplication by a scalar θ, **Methods**) preserves the stoichiometry and must not affect the simulation outcome for a correct problem formulation. However, in this case, scaling of a single reaction (RPI1) results in diverting the carbon flow from glycolysis to pentose phosphate pathway, which is one of the most important metabolic branch points (**Fig. 1**). This deviation was verified not to be consequence of alternative optima of the same mathematical solution (**Fig. S2**), thus representing different biological solutions.

**Figure 2:**
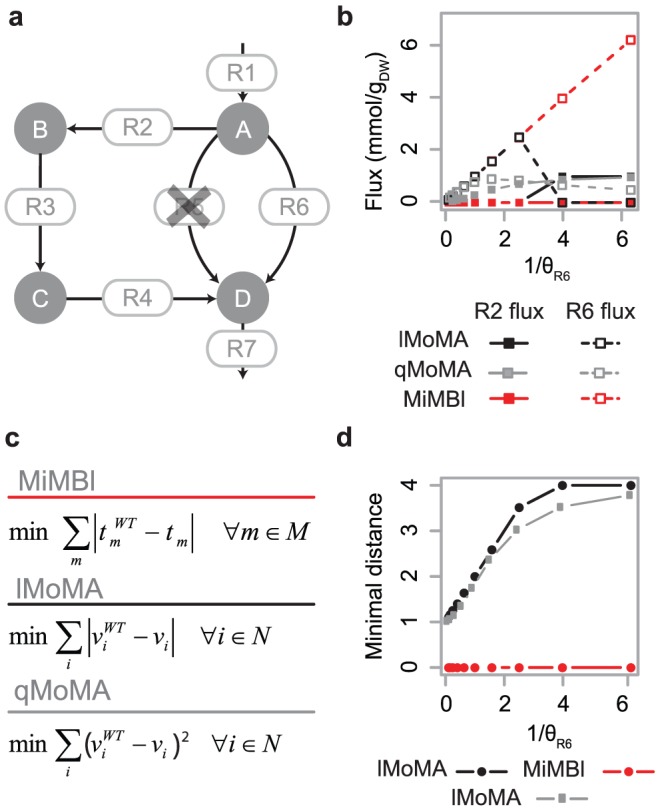
MiMBl shows robust simulation results while using alternative stoichiometry representations – illustration using a toy-model. **a**) Toy-model: *R1* to *R7* and *A* to *D* represent reactions and metabolites, respectively. In the wild-type, or reference, flux goes from *A* to *D* via *R5*. *R6* and *R2–R3–R4* are two alternative pathways for flux re-distribution after deletion of *R5*. **b**) Flux through reactions *R2* (full symbols) and *R6* (open symbols) obtained after simulation of minimization of metabolic adjustment with lMoMA (black), quadratic MoMA (qMoMA, gray) and MiMBl (red) using numerically different but biochemically equivalent representations of reaction *R6* (given by different scaling factor θ*_R6_*, **Methods**). **c**) Formulation of objective functions of minimization of metabolic adjustment for lMoMA, qMoMA and MiMBl (**Methods**). **d**) Optimal objective function value (distance) obtained for minimization of metabolic adjustment using lMoMA (black), qMoMA (gray) and MiMBl (red) as function of θ*_R6_*.

In order to provide insight into the nature of the problem leading to the susceptibility of the solution towards alternative representation of the stoichiometric matrix, we use a toy-model depicted in **Fig. 2a**. As a case study, minimization of metabolic adjustment was chosen as biological principle and formulated as minimization of Manhattan distance (a commonly used formulation of MoMA, termed lMoMA [25]). **Fig. 2** also illustrates the representation dependency of the Euclidean distance formulation of MoMA (quadratic MoMA, as originally proposed in [11]). In order to provide an intuitive insight, the following discussion is centered on lMoMA. Similar explanation holds true in quadratic space in the case of quadratic MoMA. In the wild-type toy-model, flux goes from *A* to *D* via *R5*. The goal is to predict flux distribution in the mutant lacking *R5*. The biological principle of minimization of metabolic adjustment dictates rewiring of the flux through *R6*. However, lMoMA found contradictory optimal solutions, *i.e.* solutions that re-route the flux via *R2–R3–R4* or *R6*, depending on the stoichiometric representation of *R6* (**Fig. 2b**). Insight into the cause of this behavior can be gained by analyzing the optimal objective function values, *i.e.* distances, as function of θ*_R6_* (**Fig. 2d**). Smaller θ*_R6_* implies higher numerical value of the flux through *R6*, hence higher contribution of *R6* to the distance. Consequently, after a certain value of θ*_R6_*, the activation of the longer *R2–R3–R4* pathway more than compensates the use of *R6*. The two solutions are not alternative optima, as the objective function value neither remains constant nor linearly scales with θ*_R6_*. Such non-linear dependency of the objective function value on the scalar θ*_R6_* violates the requirement of a correct problem formulation. Indeed, we analytically demonstrate that the optimality condition for the linear programming problem after scaling is not guaranteed to be satisfied in the case of using sum of fluxes as part of the objective function (**Methods**). Notably, widely used FBA-like problems (max/minimization of a single flux) are perfectly robust concerning the scaling of the stoichiometric coefficients. As a single flux is used in the objective function, the relative values of all the remaining fluxes (which depend on the stoichiometry representation) does not influence the optimal solution to be found (for a theoretical proof, see **Text S2**).

The mathematical caveat illustrated above means that the contribution of the desired biological objective function towards the obtained solution is inseparable from that of the artifacts of stoichiometry representation. Importantly, in large metabolic networks the effects of stoichiometric representation of reactions are cumulative. As we herein show, this problem can be solved by proper normalization of the objective function variables with respect to stoichiometric representation of the reactions. To achieve such normalization, we devised two approaches, normalized lMoMA (normlMoMA) and Minimization of Metabolites Balance (MiMBl). In normlMoMA, each variable in the objective function is normalized by its value in the wild-type flux distribution. Albeit being simple, this normalization method has three major drawbacks: i) many reactions often have null fluxes in the wild-type, thus posing a problem for normalization (**Methods, Text S2** and **Fig. S3**); ii) it requires a reference flux distribution to obtain the normalization factors, making it inappropriate to formulate objective functions such as minimization of overall intracellular flux; and iii) the influence of each flux on the metabolic adjustment would be exclusively due to its fold change, not taking into account that reactions carrying higher fluxes could have a stronger impact on the predicted flux distribution, as implied in the original concept of minimization of metabolic adjustment.

### Minimization of Metabolites Balance - MiMBl

To obtain a biological meaningful and mathematically robust normalization, we propose Minimization of Metabolites Balance (MiMBl) as a new method for metabolic modeling. The objective function in MiMBl is formulated as a linear combination of metabolite turnovers (t_M_). The turnover of a metabolite is the sum of all fluxes producing (or consuming) it, multiplied by the corresponding stoichiometric coefficients (**Methods**). The objective function for minimization of metabolic adjustment is reformulated to include metabolite turnovers instead of fluxes (**Fig. 2c**). Because the stoichiometric coefficients are taken into account while calculating t_M_, MiMBl is robust to the linear scaling of the stoichiometric matrix, analytical proof of which is presented in the **Methods** section. In case of the toy-model (**Fig. 2a, d**), this robustness is illustrated by the invariant nature of the objective function as well as the flux distribution. Note that the flux through R6 linearly scales with θ*_R6_*, while the turnover of all metabolites is conserved. The normalization implied in MiMBl formulation is suitable for addressing a variety of biological questions involving different objective functions, such as minimization of overall intracellular flux (by using a null vector for wild-type flux distribution) or maximization of ATP yield (by maximizing the ATP turnover for a given substrate uptake rate), among others (**Table 1**).

**Figure 3:**
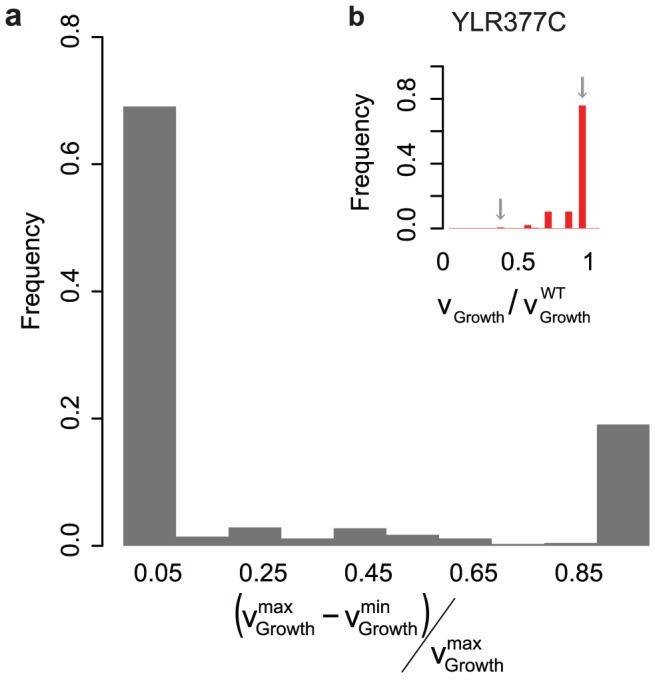
Sensitivity of MiMBl towards the use of alternative reference flux distributions. **a**) The histogram shows the distribution of variability in the predicted growth of single gene knockout mutants while using 500 different FBA alternative optima as reference flux distributions. **b**) Case study of *YLR377C* knockout simulations using different reference flux distributions as input. The predicted growth varies between 50–100% of that of the wild-type.

While mapping the flux space into the metabolite space for the objective function formulation, as we do for MiMBl, it is possible that, for a few cases, alternative flux distributions are found around a given metabolite. We therefore introduce a second optimization step that reinforces the proximity to the reference flux distribution. This is achieved by using a normlMoMA routine where the optimal objective function value found in the first MiMBl optimization step is used as an additional constraint (**Methods**). Nevertheless, highly connected metabolites ensure a degree of network connectivity, which is sufficient for decreasing the number of situations where alternative flux distributions around the same metabolite are picked by MiMBl. Indeed, we did not find any case in the simulations performed for this study where growth prediction was altered in the second optimization step. An example case where the second optimization step will be more relevant is simulations involving export of metabolites, where the choice of a particular transporter (as in the reference flux distribution) among several alternative options is desired. A more thorough analysis of MiMBl alternative optima in a genome-scale network is presented below (**Fig. 3**).

### Lack of stoichiometric normalization confounds biological interpretation

In order to estimate the extent to which the lack of normalization of stoichiometric coefficients within the objective function influences the biological interpretation of simulation results, we used lMoMA for simulating gene knockouts in the *S. cerevisiae* genome-scale metabolic model *i*FF708 [21]. In case of single gene knockout, three genes were found to change their status from non-essential to essential while using two biochemically equivalent matrix representations (**Table S1**). For instance, the mutant lacking *YCR012W*, coding for a 3-phosphoglycerate kinase (pPGK1), was predicted to be viable when using the *as-published* representation of the stoichiometric matrix S_0_ [21], and non-viable while using the biochemically equivalent matrix S_1_ (**Methods**). Based on such contradictory results, conclusions cannot be taken on whether *YCR012W* is predicted to be essential or not. As the number of deleted genes (or other network perturbations) increases, cumulative phenotypic effects related to the functional interactions between the genes are expected to take place and examples as the one mentioned above become even more striking. For triple gene knockouts, more than 200,000 triplets were found such that their predicted phenotype switched from lethal to non-lethal (or vice-versa) for the two biochemically equivalent matrix representations (**Table S1**). From a biotechnological perspective, predictions from genome-scale modeling have direct influence on the choice of gene targets selected for metabolic engineering. By using lMoMA, we identified metabolic engineering strategies (by simulating all possible combinations of knockouts of up to three genes, **Text S1**) for production of two different compounds in yeast: succinate – a native product, and vanillin-glucoside – a heterologous product. Not only a significant fraction of mutants had divergent predictions for product yield when using two biochemically equivalent stoichiometric matrices, but also several highly ranked strategies in one case were low priority targets in the other (**Figs. S4, S5, S6, S7**). Moreover, we also observed that the number of predicted synthetic lethal pairs differed by more than two-fold when using alternative stoichiometric matrix representations (**Table S2**). These inconsistencies have immediate implications on the consequent biological interpretation, as well as on the experimental design, and can be successfully overcome by using MiMBl (**Fig. S4, Table S3**).

### Alternative optima and sensitivity towards reference flux distribution

The above analysis proved the robustness of MiMBl towards stoichiometric representation of metabolic reactions. However, some degree of uncertainty in the simulation results might still exist, as we shall show here, essentially arising from two main sources (**Fig. S8a**): i) sensitivity of the results towards the initial wild-type flux distribution used as input for minimizing the metabolic distance; and ii) potential non-uniqueness of the linear programming solution while simulating the mutant phenotype, *i.e.* existence of alternative optima. The sensitivity analysis for MiMBl towards both sources of uncertainty was performed using *i*AZ900 reconstruction of the yeast metabolic network, as the same reconstruction is subsequently used to study genetic interactions within the yeast metabolism. Both sources of variability also have impact on lMoMA simulation results (**Fig. S9**).

Firstly, we analyzed the sensitivity towards the wild-type (or reference) flux distribution used as input for minimization of metabolic adjustment. Using an accurate reference flux distribution is crucial for obtaining biologically meaningful simulation results. While some metabolite exchange rates are commonly available as experimentally derived constraints for the wild-type, they are usually not sufficient to uniquely estimate the corresponding intracellular fluxes, *e.g.* by using FBA (**Figs. S8a and S1**). It has been previously shown that the use of alternative optima within the reference flux distribution obtained with FBA can affect the prediction of growth upon gene deletions using quadratic MoMA [11]. We herein performed a similar analysis by using MiMBl. The growth of single gene deletion mutants was simulated with MiMBl while using alternative optimal FBA flux distributions as reference (**Methods**). Similarly to what was previously observed for quadratic MoMA [11], cases were found where the use of alternative FBA flux distributions, as input to MiMBl, influences the growth prediction (**Fig. 3**). 70% of the predictions of single gene deletion phenotypes were consistent across all FBA-alternative-optima used, while the remaining 30% showed dependence on the input reference flux distribution. Use of additional experimentally determined constraints, for instance as obtained with ^13^C flux analysis, will be useful for reducing the uncertainty in the input flux distribution and thereby in obtaining more robust predictions.

In order to assess the variability due to potential non-uniqueness of the optimal solution obtained with MiMBl (**Fig. S8a**), we performed a flux variability analysis [26]. Biologically, the alternative optima correspond to the existence of alternative pathways that result in equivalent mutant phenotypes with regards to the required metabolic adjustment. For a fixed reference flux distribution, we calculated the range of variability of intracellular fluxes upon constraining the metabolic adjustment (*i.e.* sum of metabolite turnover distance) to its optimal value (**Methods**). All of the tested fluxes were observed to have very low or no variability (v_i_^min^/v_i_^max^>0.99) across all single gene deletion phenotypes. Utility of the second step of MiMBl was seen in case of the flux through *PGM1* upon deletion of *YOR128C* (**Fig. S8b**). Nevertheless, existence of a unique solution is problem dependent and it should be verified whether the possibility of alternative optima affects the prediction of fluxes of interest. Therefore, we performed an exhaustive analysis of variability of growth prediction across all single gene deletions, as well as all double gene deletions included in the genetic interactions case study. Growth was uniquely predicted in all these cases (**Fig. S8c**).

### Predicting genetic interactions by using MiMBl

To what extent MiMBl contributes for increasing biological understandings gained from the application of optimization-based metabolic modeling? To address this question, we used one of the most recent and comprehensive *S. cerevisiae* models, *i*AZ900 [22], to run simulations for single and double gene knockouts and challenged MiMBl to predict the epistasis scores of all significantly interacting non-essential gene pairs reported by Szappanos *et al.* [2]. Genetic interaction networks are valuable resources towards deciphering the complex genotype-phenotype relationships. A genetic interaction between two genes occurs when the phenotype displayed by a double deletion mutant is different than the one expected based on the phenotypes of the single mutants. Accordingly, two genes can display positive, negative or no interaction. In order to capture most of the biological information contained in the experimental dataset, we used two different objective functions, maximization of growth (FBA) and minimization of metabolic adjustment (MiMBl). FBA is expected to cover situations where maximization of growth is the cellular objective, while MiMBl will account for regulatory effects inherent to the wild-type flux distribution, in the sense that the flux distribution in the perturbed network is kept as close as possible to that of the wild-type. This principle of proximity to the wild-type (or the reference) should partially reflect principles of flux reorganization in genetically perturbed networks. Although, both FBA and MiMBl performed equally well concerning gene essentiality predictions (∼60% sensitivity, **Text S1**), the benefit of using MiMBl as a biological objective function became apparent while predicting genetic interactions. This implies that the biological regulatory principle underlying MiMBl is manifested in yeast (under the investigated conditions) at larger network perturbations or less drastic phenotypes than essentiality. When applied for studying genetic interactions, FBA is a conservative method compared to MiMBl, since the parameter used to define and measure genetic interactions is also the objective of optimization, *i.e.*, growth. Within the metabolic network, the existence of several optimal solutions theoretically satisfying maximum biomass formation is often observed. In case of a single/double gene deletion mutant where an alternative optimal pathway exists, FBA will always find such an alternative solution, even though it may not be biologically plausible due to regulatory constraints, and, thereby may miss potential genetic interactions. On the other hand, MiMBl will help in capturing more refined regulatory effects where the loss of growth is a side effect of minimizing the flux rerouting relative to the wild-type.

**Figure 4:**
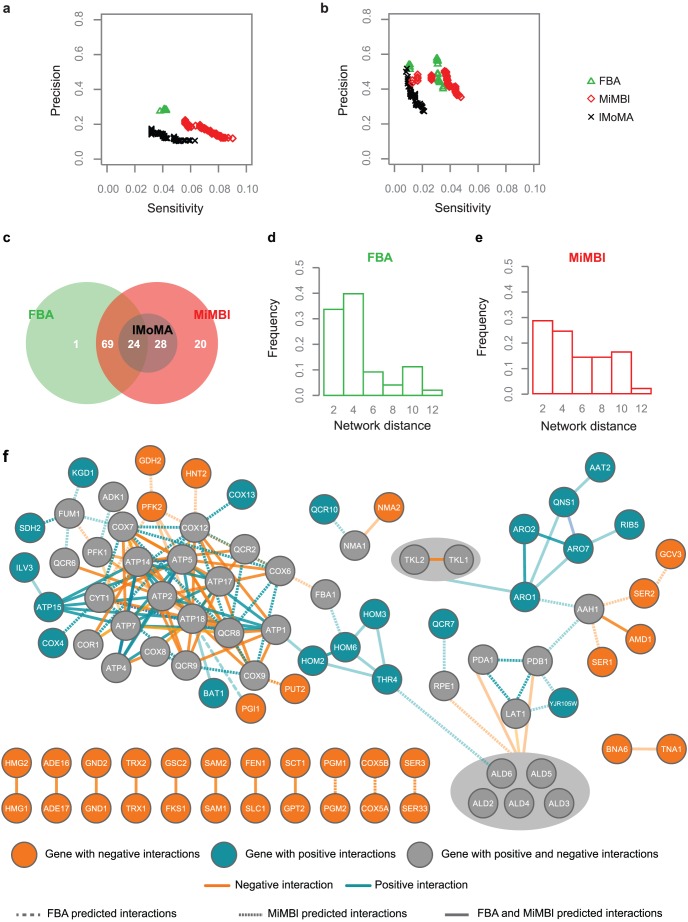
Understanding genetic interactions by using MiMBl. **a, b**) The accuracy of genetic interaction predictions by FBA, lMoMA and MiMBl was assessed by calculating the sensitivity and precision for positive (a) and negative (b) interactions. Sensitivity was calculated as the fraction of experimentally observed interactions captured by the algorithm, while precision was estimated as the fraction of experimentally observed interactions among the predicted interactions. **c**) Venn diagram showing the overlap of the correctly predicted interactions by FBA, MiMBl and lMoMA. **d, e**) Distribution of the graph theoretical distances, within the yeast metabolic network, between the interacting genes captured by FBA (d) and MiMBl (e). As MiMBl also captured the majority of FBA predicted interactions, only exclusive MiMBl interactions are shown in (e). **f**) The *S. cerevisiae* genetic interactions network correctly predicted by MiMBl and/or FBA (FBA – dashed line, MiMBl – dotted line, both – full line). Positive and negative interactions are distinguished by color (orange and blue, respectively) and the opacity of the edges is inversely proportional to the network distance between the corresponding genes. Gray-filled nodes represent genes that display both positive and negative interactions. Gray areas enclose isoenzymes where at least one of them was found to interact with other genes in the metabolic network.

The subset of experimental genetic interactions involving non-essential genes from the yeast metabolic model contains 2745 interactions (939 positive and 1806 negative) connecting 520 genes (**Text S1, Table S4**). In order to assess the performance of the different algorithms, we carried out a sensitivity *versus* precision analysis. Precision was calculated as the fraction of experimentally validated interactions among all predicted interactions, while the sensitivity represents the fraction of the experimentally validated interactions captured by the predictions (**Text S1**). A computational epistasis score cutoff (ε_cutoff_) was used to call a particular gene pair to be positively interacting (ε>ε_cutoff_), negatively interacting (ε<−ε_cutoff_) or non-interacting (|ε|<ε_cutoff_) (**Text S1**). The performance of all algorithms (MiMBl, FBA, lMoMA and quadratic MoMA) is summarized as ROC (partial receiver operating characteristic) curves for both, positive and negative epistasis (**Fig. 4** **a, b and Fig. S10**). The sensitivity and precision of the FBA predictions obtained in this study are within the same range as previously reported by Szappanos *et al.* [2]. MiMBl shows less precision than FBA in case of both positive (∼20% and ∼30%, respectively) and negative interactions (∼50% and ∼60%, respectively), but its sensitivity is considerably higher in both cases (∼9% *vs* ∼4% for positive, **Fig. 4a**; ∼5% *vs* ∼3% for negative, **Fig. 4b**), which reflects the conservative nature of FBA in predicting genetic interactions. Notably, for the entire range of genetic interaction cutoffs, MiMBl sensitivity and precision are considerably higher than those of lMoMA (**Fig. 4a, b**). The same trend was verified when the originally proposed quadratic MoMA formulation was used (**Fig. S10**). As previously reported by Szappanos and co-workers, lMoMA does not improve FBA predictions. This observation further emphasizes that a proper mathematical formulation of the biological principle (objective function) has a major impact on the ability to interpret *in vivo* observations.

We chose a strict interaction cutoff (|ε_cutoff_| = 0.013) for further analysis of the predicted interactions (**Fig. S11**). For this cutoff, the correctly predicted genetic interactions map contains 142 interactions (73 positive and 69 negative) connecting 86 genes (**Fig. 4f**). MiMBl not only captures all interactions, except one, predicted by FBA, but also contributes with 48 additional interactions (∼34% of all accurate predictions). MiMBl predictions thus span almost all of those from FBA (**Fig. 4c**), which we attribute to the fact that many metabolites within the metabolic model are directly contributing to the biomass formation. Consequently, if the turnover of most metabolites is kept constant upon gene deletions, the biomass turnover (growth) will also remain constant. On the other hand, FBA is not able to capture many genetic interactions found by MiMBl (**Fig. 4c**). These will involve mutants where the loss of fitness upon gene deletion is caused by the change from an *in vivo* well-tuned pathway to an alternative pathway containing different metabolites or enzymes. For many of such cases, there are alternative pathways that sustain the same growth as the reference and FBA finds such solutions, regardless of the magnitude of the turnover adjustment that is required for the cell. Because of this feature, MiMBl is capable of capturing a part of the regulatory constraints on the operation of cellular metabolism, which lMoMA failed to capture (**Fig. 4c**).

The regulatory constraints imposed by MiMBl assume even stronger relevance in the case of positive interactions, where MiMBl exclusively accounts for almost 50% of all successfully predicted interactions (**Fig. 4f**). In fact, FBA's ability of predicting positive interactions is limited, as the maximum predicted biomass formation of a double deletion mutant would never be higher than the highest among those predicted for the two single deletion mutants. Thus, if a single deletion mutant has the maximum predicted fitness of 1 (meaning that the fitness of the mutant is the same as that of the wild-type), positive interactions involving the deleted gene will be impossible to predict. As FBA is bound to find the optimal solution that provides the highest growth, single mutants with maximum fitness are much more often predicted than the ones found by MiMBl, where minimal adjustment of the metabolic network is preferred over maintaining maximum growth. Indeed, MiMBl predicts decreased single mutant fitness for twice more gene knockouts than FBA (∼38.4 *vs* 18.1%). Consequently, MiMBl also displayed an improved capacity to predict both positive and negative epistasis involving the same gene. More than 80% of the genes display this feature *in vivo*. Interestingly, 30% of the genes involved in MiMBl predicted epistasis interact both positively and negatively, while FBA predicts that only 14% of the genes do so (**Fig. 4f**).

### MiMBl predicts genetic interactions between distant genes in the network

As metabolic networks are featured by several metabolites with a high degree of connectivity, interactions occur between distant pathways in the network. To assess MiMBl's ability to predict such pleiotropic effects, we calculated the network distance between each pair of genes accurately predicted to interact (**Text S1**). MiMBl captured interactions between genes that are significantly more distant than in case of FBA (∼40% more distant for negative epistasis, p-value = 0.022; ∼10% more distant for both positive and negative epistasis, p-value = 0.089; **Fig. 4d, e**).

### Predicting genetic interactions of isoenzymes

In a metabolic network reconstruction, a group of isoenzymes is represented by a single reaction, which is associated with two or more genes. Simulation-wise, such a reaction will be inactive only when all of the corresponding isoenzyme-coding genes are deleted and deletion of any single gene will not result in a loss of fitness. Thus, in case of a reaction with two isoenzymes, when the deletion of both isoenzyme-coding genes leads to decreased fitness *in silico*, a negative interaction will be predicted. Our analysis captured several of such cases, for example, the negative interactions between *SER3* and *SER33*, as well as between *SAM1* and *SAM2* (**Fig. 4f**). While this gene-deletion-centered approach allows capturing interactions between isoenzyme-coding genes, it is not suited for predicting interactions between two functionally different genes where one (or both) of them have isoenzymes. However, such interactions are often observed *in vivo*, since isoenzymes do not always completely compensate each other's function due to differences in kinetic and/or regulatory characteristics. Although these effects cannot be directly captured using the currently available metabolic modeling tools, we suggest evaluating the metabolic basis of genetic interactions between functionally different genes with isoenzymes by taking a reaction-centered approach. For this purpose, flux through reactions catalyzed by isoenzymes was constrained to zero when at least one of the isoenzyme-coding genes was deleted. This way, five additional genetic interactions involving isoenzymes were correctly captured: a positive interaction between the isoenzyme group *TLK1 & TLK2* and the gene *ARO1*, as well as four negative interactions involving the isoenzyme group *ALD2-ALD6* and other genes from the central carbon metabolism (**Fig. 4f**). These five interactions are thus likely to result from flux rerouting caused by the lack of compensation by the corresponding isoenzymes.

### Combining MiMBl and FBA predictions for understanding genetic interactions

Use of MiMBl not only allowed us to expand the range of genetic interactions predicted by FBA, but also the combined use of these two complementary algorithms enabled finding of relevant interactions where only one or both simulation principles apply. For example, the interaction between *PGK2* and *GDH2*, exclusively captured by MiMBl, is due to balancing of NADH and glutamate, two of the most connected metabolites in the network. As there are alternative pathways for fulfilling NADH and glutamate requirement (despite implying higher metabolic adjustments), FBA could not capture this interaction. A similar effect is observed for the negative interaction between isoenzymes *SER3* and *SER33*. In the absence of both genes, FBA predicts the needed supply of serine to be totally fulfilled by rerouting the metabolic fluxes via the glyoxylate shunt and threonine biosynthesis. On the other hand, MiMBl predicts that the supply of serine will be shared between the two alternative pathways, but the rescue cannot be complete, because the corresponding metabolic adjustment cost overweighs the benefit of increased growth. This prediction is in very good agreement with the experimental verification that the double mutant growth is impaired and can be restored by adding glycine to the medium, which is the intermediate for serine synthesis via glyoxylate or threonine [27].

Overall, our results demonstrate that the use of optimization-based algorithms that are stoichiometry representation independent is fundamental for unambiguously linking modeling results with biological interpretation. To this end, we report a new method for formulating objective functions for metabolic modeling – MiMBl. As a biological case study, we used MiMBl to gain insights into the flux rewiring underlying genetic interactions within the yeast metabolic network. The analysis showed that the combined use of different objective functions is of primary importance in order to achieve a more complete understanding of the operating principles behind complex biological phenomena such as genetic interactions. Indeed, the number of accurately predicted genetic interactions was almost doubled owing to the use of MiMBl, highlighting the impact of metabolic adjustment constraints on the operation of perturbed metabolic networks. In conclusion, MiMBl provides a framework for consistent mathematical formulation of biological objective functions and thereby facilitates unraveling of the genotype-phenotype relations in metabolic networks.

## Methods

### Yeast genome-scale metabolic reconstructions

The susceptibility of the modeling results towards the stoichiometry representation is inherent to the formulation of the objective function; and it is independent of the choice of metabolic network reconstruction. Two reconstructions were therefore selected in this study based on their suitability for addressing the biological principle in question. *i*FF708 [21] was the reconstruction of choice for illustrating the prediction of internal flux distribution and metabolic engineering targets, as i) this reconstruction has been successfully used for model guided metabolic engineering [13], [14] and, ii) when constrained with experimentally measured substrate and product exchange rates [28] (**Text S1**), *i*FF708 showed less flux variability at physiologically important flux nodes as opposed to more recent reconstruction *i*AZ900 [22] (**Fig. S1**). On the other hand, for studying large-scale genetic interactions in yeast, we used *i*AZ900 (manually curated from iMM904 [29]), as the maximum gene coverage overlap with the experimental dataset was the main criterion. Simulation conditions are provided in **Text S1**.

### Normalized lMoMA

Normalized lMoMa was formulated as follows:
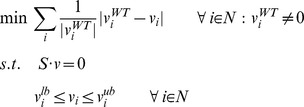
Where *N* is the set of all reactions, *M* is the set of all intracellular metabolites, *S* is the stoichiometric matrix and *v*_i_ is the flux for reaction *i*. *WT* stands for wild-type (or reference), *v_i_^lb^* and *v_i_^up^* are the lower and upper bounds for the flux of reaction *i*.

### Minimization of metabolites balance – MiMBl

Metabolite turnover is defined as the sum of all fluxes producing (or consuming) it, multiplied by the stoichiometric coefficients:
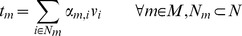
N_m_ is the subset of N producing or consuming metabolite *m* and α*_m,i_* is the stoichiometric coefficient of metabolite *m* in reaction *i*. Note that α*_m,i_* is always a positive number in the definition above, irrespective of *m* being a substrate or a product.

MiMBl was formulated as two sequential linear programming problems, as follows:

1^st^ optimization:
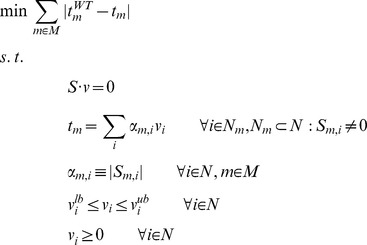

2^nd^ optimization:
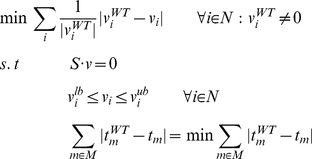

We note that MiMBl integrates reaction-to-metabolite turnover mapping into the model formulation in terms of defining biological objective functions and thereby making metabolite-usage a determinant for the prediction of metabolic phenotypes. This formulation is thus different from metabolite-centric approaches that have been proposed for interpreting simulation results [30]–[32].

### Alternative stoichiometry representations

Alternative stoichiometry representations were obtained by multiplying a given reaction (or a set of reactions) by a scalar θ (or a set of scalars). Consider reaction r 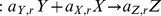, for which an equivalent representation is given by:
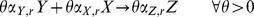
where *Y, X* and *Z* represent the metabolites participating in reaction *r* and *α_Yr_, α_Xr_, α_Zr_* represent the corresponding stoichiometric coefficients. Note that when the stoichiometry of reaction *r* is scaled by θ, the corresponding flux value will be scaled by 1/θ for the same optimal solution. For illustrating the impact of linear scaling of the reactions stoichiometry on the internal flux distribution, the reaction *RPI1* of *i*FF708 model was divided by the scalar θ. For illustrating the impact of using alternative stoichiometry representations on the design of metabolic engineering strategies, two biochemically equivalent stoichiometric matrices were used: i) the as-published matrix from the yeast model (*S_0_*) and ii) an equivalent matrix (*S_1_*) where the stoichiometric coefficients of the reactions *SERxtO, PDC6, FUR1, GAP1_21, PNP1_1*, and *CYSxtO* were divided by 100, 100, 0.1, 0.01, 100 and 0.1, respectively. A third equivalent matrix (*S_2_*) was generated by dividing the coefficients of a single reaction (*PGK1*) by 0.1. The results of the comparison between *S_0_* and *S_2_* are presented in **Fig. S6**.

### Impact of scaling stoichiometry on the optimal solution – Analytical evidence

The impact of scaling the constraints of a given linear programming problem depends on whether such changes guarantee the optimality conditions after scaling. Consider the problem:
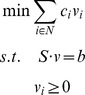
Where 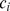 is the cost coefficient of variable 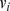 in the objective function. Here, a linear combination of non-normalized fluxes is used in the objective function, similarly to *e.g.* minimization of intracellular flux and lMoMA. Assuming that 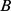 is an optimal basis matrix for the problem, the following optimization condition is satisfied:
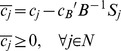
where 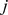 is the index of variable 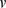 in matrix 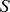, 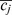 is the reduced cost of the variable 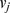, 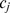 is the objective function coefficient of 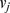, 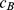 is the vector containing the objective coefficients of basic variables and 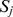 is the *j^th^* column of matrix 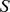 [33]. Linear scaling the problem by the matrix 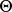 will result in the following reduced cost for each variable:
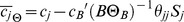
Where 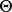 is a 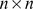 positive diagonal matrix (**scaling matrix**) and 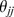 is the scaling factor for the *j^th^* column of matrix 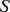. In the cases of entries 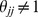 the corresponding columns of 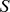 are accordingly scaled. Analogously, 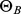 is the scaling matrix corresponding to the basic variables.

Unless all entries of 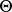 are identical,
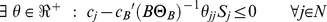
Therefore the **optimality condition is not guaranteed**.

**Corollary 1**: When all (diagonal) entries of 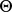 are identical (uniform scaling matrix), and therefore equal to 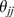, the optimality condition is simplified to
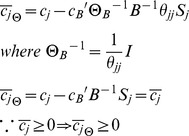
The same optimality condition can thus be guaranteed only when the matrix 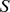 is uniformly scaled. Note that due to the nature of the biological problem, the genuine representation of 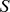 might not be known, thereby 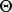 cannot be guaranteed to be a uniform scaling matrix. More importantly, for metabolic modeling purposes (where flux units and ranges are problem dependent), it is nevertheless undesirable that the solution is sensitive to non-uniform scaling and thus context dependent.

**Corollary 2**: For any positive diagonal scaling matrix 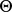, the same optimality condition is still guaranteed if the cost coefficients vector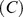 is also scaled by 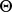. However, the choice of the appropriate 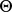 for formulating a biologically meaningful problem will require existence of a unique representation of 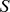 for any given network, which is not possible due to subjective nature of stoichiometry representation.

Now consider the following MiMBl-like formulated problem:
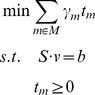
Where, 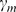 is cost coefficient of variable 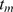 in the objective function. The new problem biologically corresponds to the previous one, after mapping the flux space into metabolite space. We term it as a MiMBl-like problem formulation.
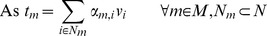
Recall that 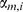 is the stoichiometric coefficient of metabolite 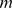 in reaction 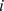. The objective function can be re-written as function of 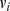:
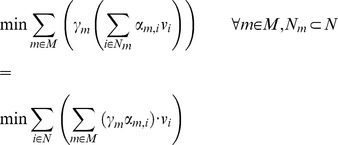
Therefore, the objective function coefficient of each 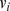 is a function of the stoichiometric coefficients 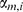: 
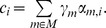


Similarly to the previous problem, the following optimality condition is satisfied, so 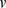 is an optimal solution.
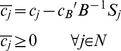
Scaling the optimality condition will result in:
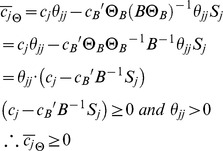
Unlike the previous situation (sum of fluxes in the objective function), using a MiMBl-like problem formulation guarantees that the optimality condition is always satisfied, independently of the stoichiometry representation.

### Sensitivity analysis

The sensitivity of MiMBl and lMoMA towards the use of FBA alternative optima for wild-type flux distribution was evaluated by performing single gene deletion simulations while using 500 different flux distributions corresponding to alternative optima of the same FBA solution. FBA alternative optimal solutions were obtained following a Mixed Integer Linear Programming (MILP) routine similar to the one suggested by Lee *et al.* [34]. Flux variability analysis of the flux distributions obtained with MiMBl and lMoMA were performed according to the procedure suggested by Mahadevan *et al.* [26]: maximizing and minimizing internal fluxes after constraining the objective function to its optimal value. In case of MiMBl, this implies adding an additional constraint of the minimum Manhattan distance between the wild-type and the mutant metabolite turnovers. In case of lMoMA, the Manhattan distance between the mutant and the wild-type fluxes will have an upper bound. Growth is uniquely predicted if 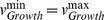. Cases of 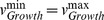 = 0 were also treated as 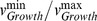 = 1, solely for the purpose of visualization (**Fig. 3d**).

## Supporting Information

Figure S1 Comparing the variability of predicted internal fluxes of glycolysis and pentose phosphate pathway between the models *i*FF708 and *i*AZ900. Metabolites uptake and production rates, as well as growth from [28] were used to constraint both models and a flux variability analysis as suggested by [26] was performed for the represented fluxes from a) glycolysis and pentose b) phosphate pathway. Flux names are represented as in *i*FF708 [21].
(TIF)

Figure S2 Profiles obtained for the objective function value (minimization of overall intracellular flux) using alternative stoichiometry representations of *S. cerevisiae* genome-scale model *i*FF708 [21]. This analysis is complementary to and based on the same simulation constraints as used for **Fig. 1** in the main text. As the contribution of each flux to the objective function changes based on the corresponding stoichiometry representation, different situations could be described, leading either to the same (a, b) or distinct (c, d) optimal solutions. To illustrate these different situations, four reactions within the model were linearly scaled one at a time by multiplying by a scalar θ as described in **Methods**. **a**) Linear scaling of the reaction *FBP1*. As *FBP1* carries no flux under the simulated conditions, the scaling of this reaction does not affect the objective function value. **b**) Linear scaling of the reaction *RPE1*. For the range of θ tested, the objective function value perfectly correlated with the scaling factor of the reaction *RPE1*, which indicates that all obtained solutions are in fact the same optimal solution (or alternative optimal solutions, depending on the model complexity). This profile means that there is no pathway alternative to *RPE1* that can become part of the optimal solution. **c**) Linear scaling of the reaction *RPI1*. For the range of tested θ, at least two slopes are observed when correlating the objective function value with 1/θ, indicating that at least two different optimal solutions were found for the same problem. **d**) Linear scaling of the reaction *NDI1*. Similarly to that of *RPI1*, scaling of *NDI1* leads to different optimal solutions. However, in this case, the objective function value stabilizes after a given θ, which means that this flux no longer influences the optimization. Such profile suggests that the optimal solution found after the given value of θ does no longer involve *NID1*, but an alternative pathway, which became preferred for minimizing the objective function.
(TIF)

Figure S3 A toy-model illustrating how, and why, alternative stoichiometry representations influence simulation of minimization of metabolic adjustment by using normalized lMoMA – normlMoMA. **a**) Toy-model: *R1* to *R7* and *A* to *D* represent reactions and metabolites, respectively. In the wild-type, or reference, flux goes from *A* to *D* via *R5*. *R6* and *R2–R3–R4* are two alternative pathways for flux re-distribution after deletion of *R5*. **b**) Flux through reactions *R2* (full symbols) and *R6* (open symbols) obtained after simulation with normlMoMA by using alternative representations of reaction *R6* (given by different θ*_R6_*, **Methods**). **c**) Formulation of normlMoMA objective function (**Methods**). **d**) Optimal objective function value (distance) obtained for minimization of metabolic adjustment as function of θ*_R6_*.
(TIF)

Figure S4 Impact of stoichiometry representation on the design of metabolic engineering strategies depending on the nature of the objective function formulation – MiMBL versus lMoMA. Shown is the comparison of predicted succinate and vanillin-glucoside yields for triple gene knockout mutants obtained with two alternative stoichiometric matrices (*S_0_* and *S_1_*, **Methods**). Number of mutants diverging in their lMoMA-predicted **a**) succinate and **b**) vanillin-glucoside yields for the two alternative representations of stoichiometry. The x-axis represents the percentage of deviation of product formation by the mutants relative to *S_0_*. **c**) Comparison of ranks of lMoMA-predicted metabolic engineering strategies for improving succinate and vanillin-glucoside production, obtained by using *S_0_* and *S_1_*. **d**) Comparison of ranks of MiMBL-predicted metabolic engineering strategies for improving succinate and vanillin-glucoside production, obtained by using *S_0_* and *S_1_*.
(TIF)

Figure S5 Stoichiometry representation impacts the design of metabolic engineering strategies for improving succinate production in *S. cerevisiae* depending on the nature of the objective function formulation. Shown is the comparison of predicted succinate yield for **a**) single, **b**) double and **c**) triple gene knockout mutants obtained with two alternative stoichiometric matrices (*S_0_* and *S_1_*, **Methods**). The number of mutants diverging in their lMoMA-predicted succinate yield for the two alternative representations of stoichiometry is represented on the y-axis, while the percentage of deviation of product formation by the mutants relative to *S_0_* is represented on the x-axis. **d–f**) Comparison of ranks of lMoMA-predicted metabolic engineering strategies for improving succinate production obtained by using *S_0_* and *S_1_* for d) single, e) double and f) triple gene knockout mutants.
(TIF)

Figure S6 Stoichiometry representation impacts the design of metabolic engineering strategies for improving vanillin-glucoside production in *S. cerevisiae* depending on the nature of the objective function formulation. Shown is the comparison of predicted vanillin-glucoside yield for **a**) single, **b**) double and **c**) triple gene knockout mutants obtained with two alternative stoichiometric matrices (*S_0_* and *S_1_*, **Methods**). The number of mutants diverging in their lMoMA-predicted vanillin-glucoside yield for the two alternative representations of stoichiometry is represented on the y-axis, while the percentage of deviation of product formation by the mutants relative to *S_0_* is represented on the x-axis. **d–f**) Comparison of ranks of lMoMA-predicted metabolic engineering strategies for improving vanillin-glucoside production obtained by using *S_0_* and *S_1_* for d) single, e) double and f) triple gene knockout mutants.
(TIF)

Figure S7 Stoichiometry representation impacts the design of metabolic engineering strategies for improving succinate and vanillin-glucoside yields in *S. cerevisiae* depending on the nature of the objective function formulation. **a–f**) Number of mutants diverging in their lMoMA-predicted a–c) succinate and d–f) vanillin-glucoside yields for two alternative representations of stoichiometry, *S_0_* and *S_2_* (**Methods**). Results for a,d) single, b,e) double and c,f) triple gene knockout mutants are presented. **g–l**) Comparison of ranks of lMoMA-predicted metabolic engineering strategies for improving g–i) succinate and j–l) vanillin-glucoside production obtained by using *S_0_* and *S_2_*. Results for g,j) single, h,k) double and i,l) triple gene knockout mutants are presented.
(TIF)

Figure S8 Alternative optima and sensitivity to reference flux distribution. **a**) The left side of the panel illustrates the variability due to possible uncertainty in the reference flux distribution, for example, as obtained by FBA simulations. The right hand side of the panel illustrates variability in the simulation result owing to the possibility of alternative optimal solutions of the MiMBL linear programming problem. Deletion of Gene 1 illustrates a case where a unique optimal solution is found, while deletion of Gene 2 depicts a case of alternative optima. **b**) Flux variability analysis to assess the existence of the alternative optimal solutions for a given reference flux distribution (Methods). Shown are the flux variability ranges of alanine transport and flux through phosphoglucomutase (PGM1) after deletion of YBL045C and YOR128C, respectively. PGM1 represents a case where the 2nd optimization step of MiMBL contributes to reducing of flux variability. The corresponding results for lMoMA are presented in Fig. S9. **c**) Flux variability analysis for growth flux following single/double gene deletions. MiMBL yielded unique growth prediction for single and double gene deletion mutants. Only double gene deletions relevant for the genetic interactions case study (main text) were simulated.
(TIF)

Figure S9Alternative optima and sensitivity to reference flux distribution: lMoMA. **a**) Sensitivity of MiMBL towards the use of different reference flux distributions (**Methods**). Shown are histograms of the simulated growth (v_Growth_/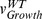) of the mutants lacking *YLR377C* or *YGL148W* obtained with MiMBL across 500 simulations using alternatively optimal FBA solutions. Gray arrows mark the minimum and the maximum ratio. **b**) Flux variability analysis to assess alternative optimal solutions for a given reference flux distribution (**Methods**). Shown are the flux variability ranges of alanine transport and flux through phosphoglucomutase (PGM1) after deletion of *YBL045C* and *YOR128C*, respectively. PGM1 represents a case where the 2^nd^ optimization step of MiMBL contributes to reducing of flux variability.(TIF)

Figure S10 ROC (partial receiver operating characteristic) curves obtained for predicting genetic interactions with MoMA. The ROC curves for the remaining algorithms were kept for reference. Sensitivity reflects the fraction of experimentally validated interactions captured by the algorithm while precision is experimentally validated interactions among all predicted interactions. **a**) Positive interactions. **b**) Negative interactions.
(TIF)

Figure S11 Sensitivity and precision for predicted genetic interactions versus epistasis score cutoff for FBA and MiMBL. The top plots present the sensitivity for positive (**a**) and negative (**b**) interactions for FBA and MiMBL. The epistasis score cutoff of |0.13| is represented by a dashed line. The bottom plots present the precision for positive (**c**) and negative (**d**) interactions for FBA and MiMBL. The epistasis score cutoff of |0.13| is represented by a dashed line.
(TIF)

Table S1 Number of lMoMA-predicted lethal gene/reaction knockouts in *S. cerevisiae* that differ between alternative representations of stoichiometry (S_1_ and S_2_), relative to S_0_.
(DOCX)

Table S2 lMoMA-predicted epistatic interactions within *S. cerevisiae* genome-scale metabolic model.
(DOCX)

Table S3 MiMBL-predicted epistatic interactions within *S. cerevisiae* genome-scale metabolic model.
(DOCX)

Table S4 All significant genetic interactions among non-essential genes from Szappanos *et al.* involving genes contained in *i*AZ900 model included in the study.
(XLSX)

Text S1 Supplementary methods. i) Yeast genome-scale metabolic models and simulation conditions; ii) Flux Balance Analysis; iii) Minimization of overall intracellular flux; iv) Minimization of metabolic adjustment – lMoMA; v) Genetic interactions – epistasis score; vi) Metabolic network distance.
(DOCX)

Text S2 Supplementary notes. i) Toy-model; ii) normlMoMA; iii) Impact of scaling stoichiometry on finding the optimal solution for metabolic flux distributions using FBA-like objective functions – Analytical evidence.
(DOCX)
