# Supplementary figures and images for: Impact of Stoichiometry Representation on Simulation of Genotype-Phenotype Relationships in Metabolic Networks
Source: PLoS Comput Biol. 2012 Nov 1;8(11):e1002758. doi: 10.1371/journal.pcbi.1002758 (PMC3486866; doi:10.1371/journal.pcbi.1002758)

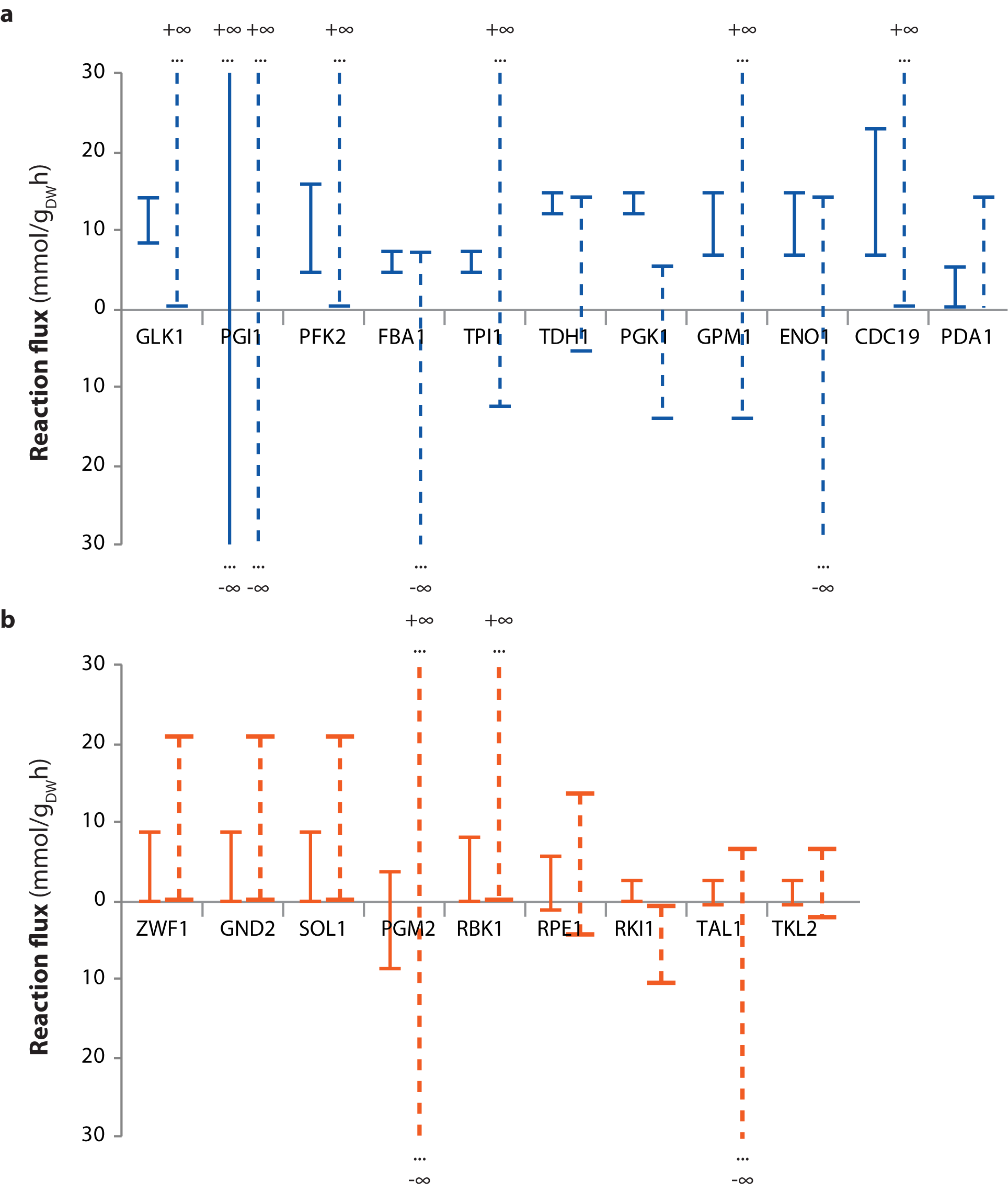

Supplement: Figure S1 — Comparing the variability of predicted internal fluxes of glycolysis and pentose phosphate pathway between the models iFF708 and iAZ900. Metabolites uptake and production rates, as well as growth from [28] were used to constraint both models and a flux variability analysis as suggested by [26] was performed for the represented fluxes from a) glycolysis and pentose b) phosphate pathway. Flux names are represented as in iFF708 [21]. (TIF) [file pcbi.1002758.s001.tif]

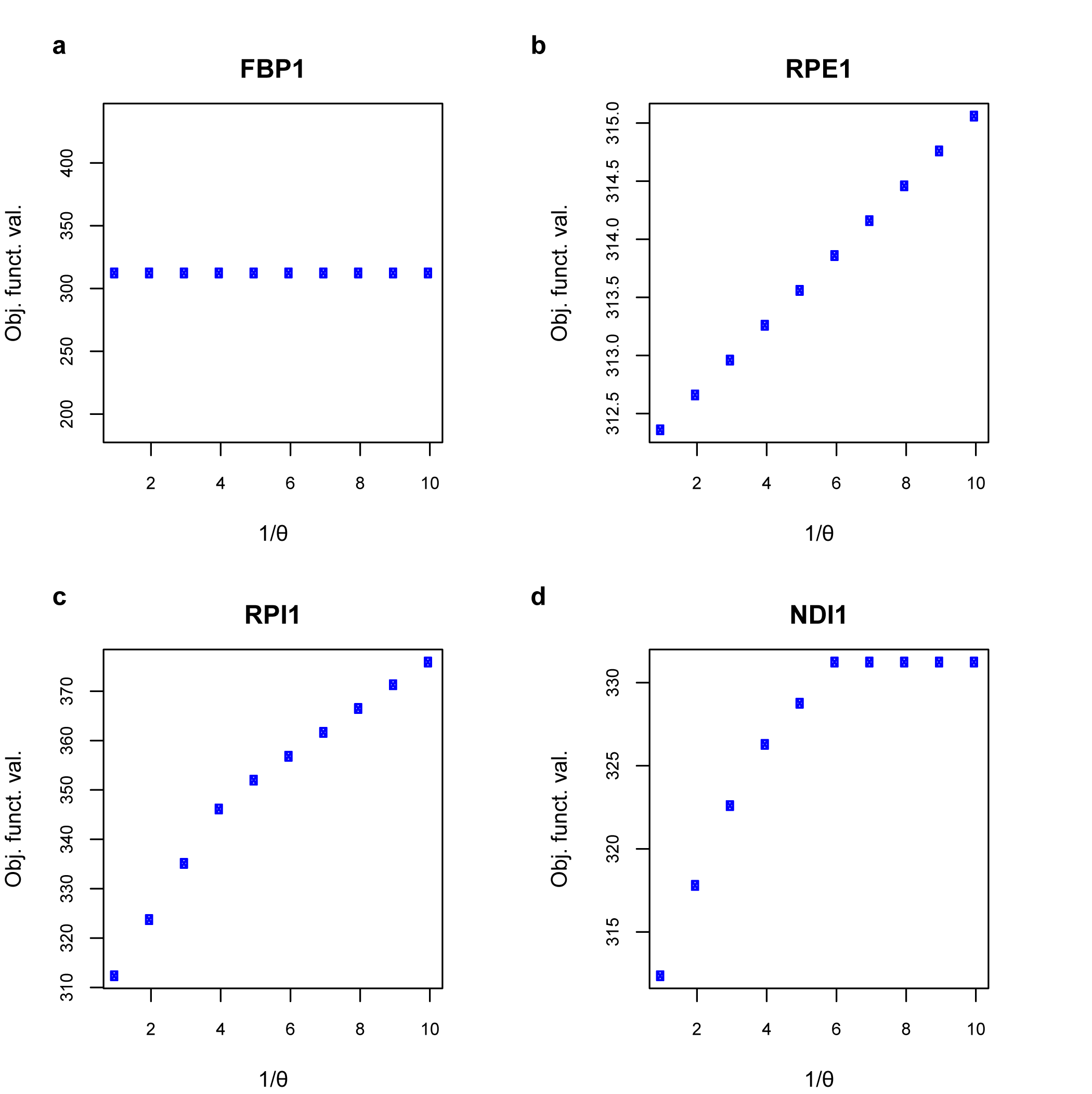

Supplement: Figure S2 — Profiles obtained for the objective function value (minimization of overall intracellular flux) using alternative stoichiometry representations of S. cerevisiae genome-scale model iFF708 [21]. This analysis is complementary to and based on the same simulation constraints as used for Fig. 1 in the main text. As the contribution of each flux to the objective function changes based on the corresponding stoichiometry representation, different situations could be described, leading either to the same (a, b) or distinct (c, d) optimal solutions. To illustrate these different situations, four reactions within the model were linearly scaled one at a time by multiplying by a scalar θ as described in Methods. a) Linear scaling of the reaction FBP1. As FBP1 carries no flux under the simulated conditions, the scaling of this reaction does not affect the objective function value. b) Linear scaling of the reaction RPE1. For the range of θ tested, the objective function value perfectly correlated with the scaling factor of the reaction RPE1, which indicates that all obtained solutions are in fact the same optimal solution (or alternative optimal solutions, depending on the model complexity). This profile means that there is no pathway alternative to RPE1 that can become part of the optimal solution. c) Linear scaling of the reaction RPI1. For the range of tested θ, at least two slopes are observed when correlating the objective function value with 1/θ, indicating that at least two different optimal solutions were found for the same problem. d) Linear scaling of the reaction NDI1. Similarly to that of RPI1, scaling of NDI1 leads to different optimal solutions. However, in this case, the objective function value stabilizes after a given θ, which means that this flux no longer influences the optimization. Such profile suggests that the optimal solution found after the given value of θ does no longer involve NID1, but an alternative pathway, which became preferred for minimizing the o [file pcbi.1002758.s002.tif]

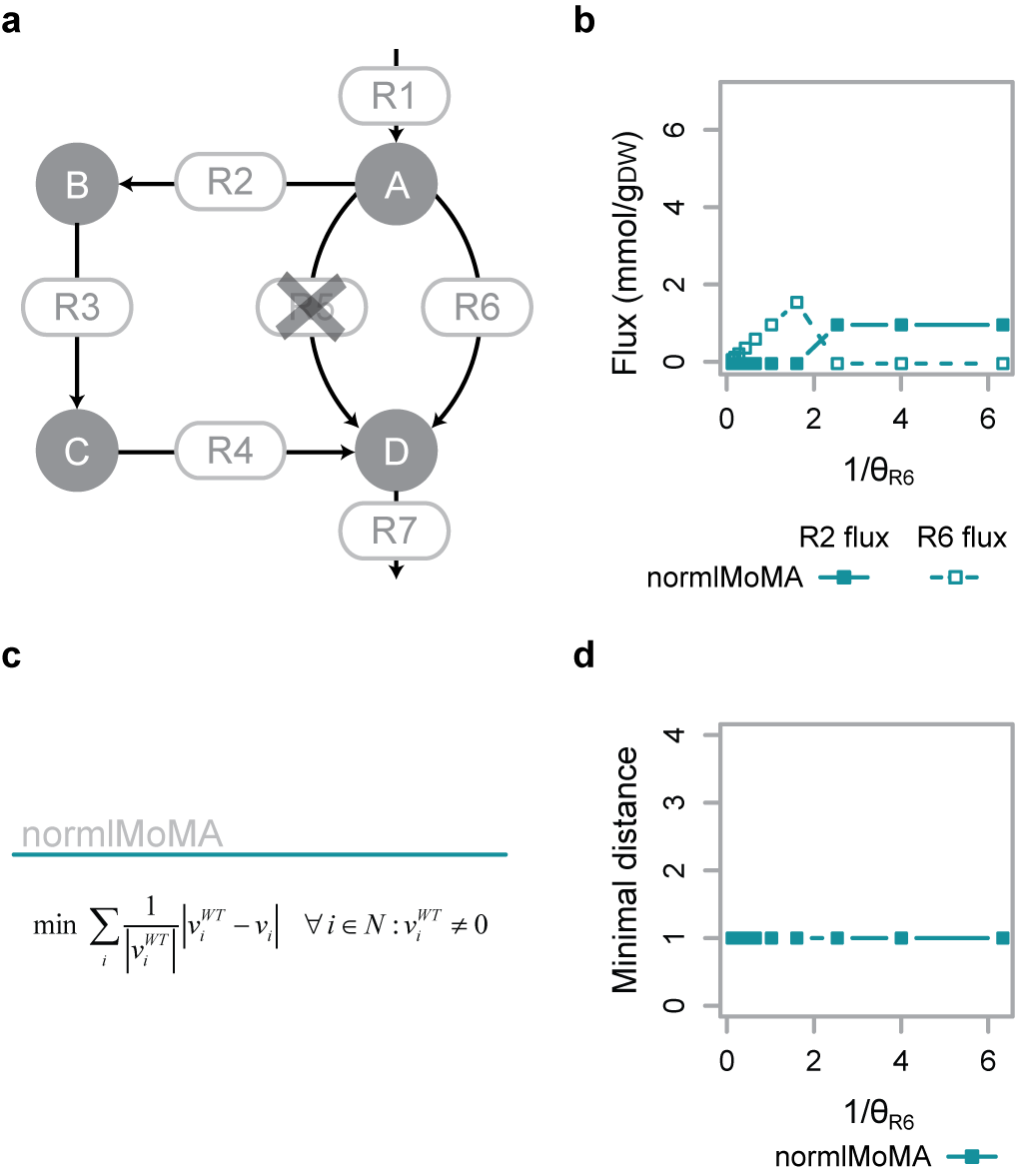

Supplement: Figure S3 — A toy-model illustrating how, and why, alternative stoichiometry representations influence simulation of minimization of metabolic adjustment by using normalized lMoMA – normlMoMA. a) Toy-model: R1 to R7 and A to D represent reactions and metabolites, respectively. In the wild-type, or reference, flux goes from A to D via R5. R6 and R2–R3–R4 are two alternative pathways for flux re-distribution after deletion of R5. b) Flux through reactions R2 (full symbols) and R6 (open symbols) obtained after simulation with normlMoMA by using alternative representations of reaction R6 (given by different θR6, Methods). c) Formulation of normlMoMA objective function (Methods). d) Optimal objective function value (distance) obtained for minimization of metabolic adjustment as function of θR6. (TIF) [file pcbi.1002758.s003.tif]

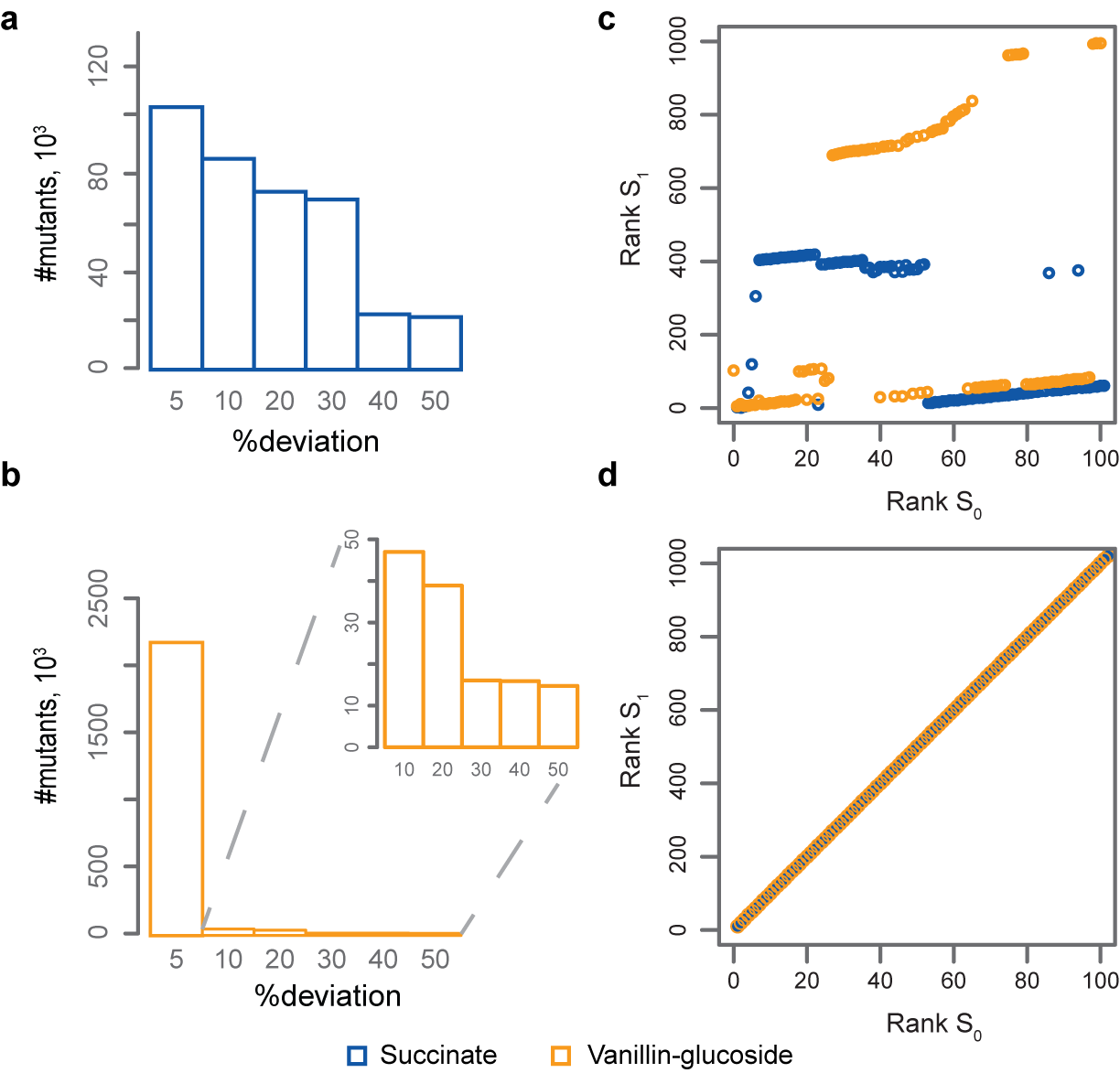

Supplement: Figure S4 — Impact of stoichiometry representation on the design of metabolic engineering strategies depending on the nature of the objective function formulation – MiMBL versus lMoMA. Shown is the comparison of predicted succinate and vanillin-glucoside yields for triple gene knockout mutants obtained with two alternative stoichiometric matrices (S0 and S1, Methods). Number of mutants diverging in their lMoMA-predicted a) succinate and b) vanillin-glucoside yields for the two alternative representations of stoichiometry. The x-axis represents the percentage of deviation of product formation by the mutants relative to S0. c) Comparison of ranks of lMoMA-predicted metabolic engineering strategies for improving succinate and vanillin-glucoside production, obtained by using S0 and S1. d) Comparison of ranks of MiMBL-predicted metabolic engineering strategies for improving succinate and vanillin-glucoside production, obtained by using S0 and S1. (TIF) [file pcbi.1002758.s004.tif]

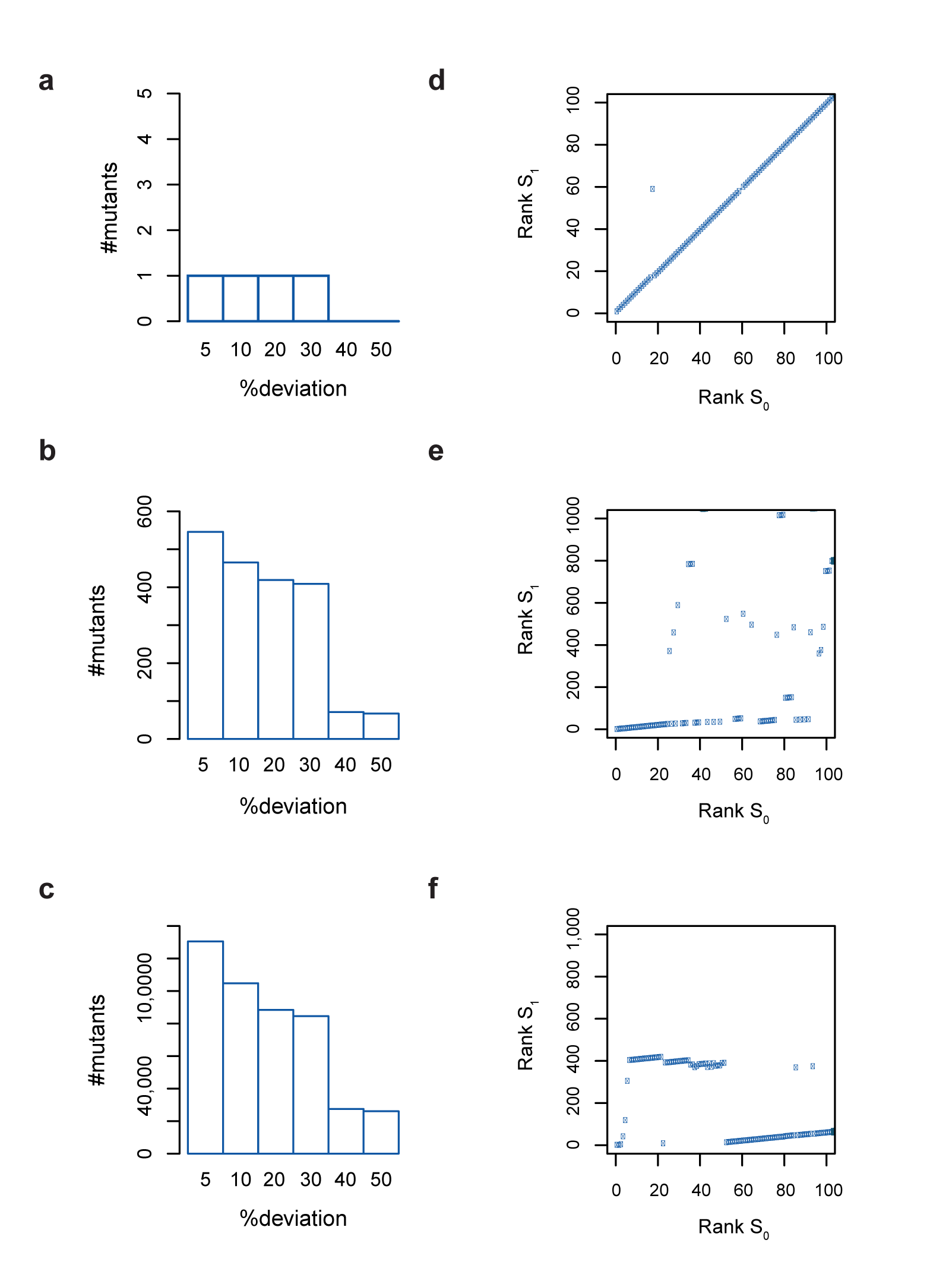

Supplement: Figure S5 — Stoichiometry representation impacts the design of metabolic engineering strategies for improving succinate production in S. cerevisiae depending on the nature of the objective function formulation. Shown is the comparison of predicted succinate yield for a) single, b) double and c) triple gene knockout mutants obtained with two alternative stoichiometric matrices (S0 and S1, Methods). The number of mutants diverging in their lMoMA-predicted succinate yield for the two alternative representations of stoichiometry is represented on the y-axis, while the percentage of deviation of product formation by the mutants relative to S0 is represented on the x-axis. d–f) Comparison of ranks of lMoMA-predicted metabolic engineering strategies for improving succinate production obtained by using S0 and S1 for d) single, e) double and f) triple gene knockout mutants. (TIF) [file pcbi.1002758.s005.tif]

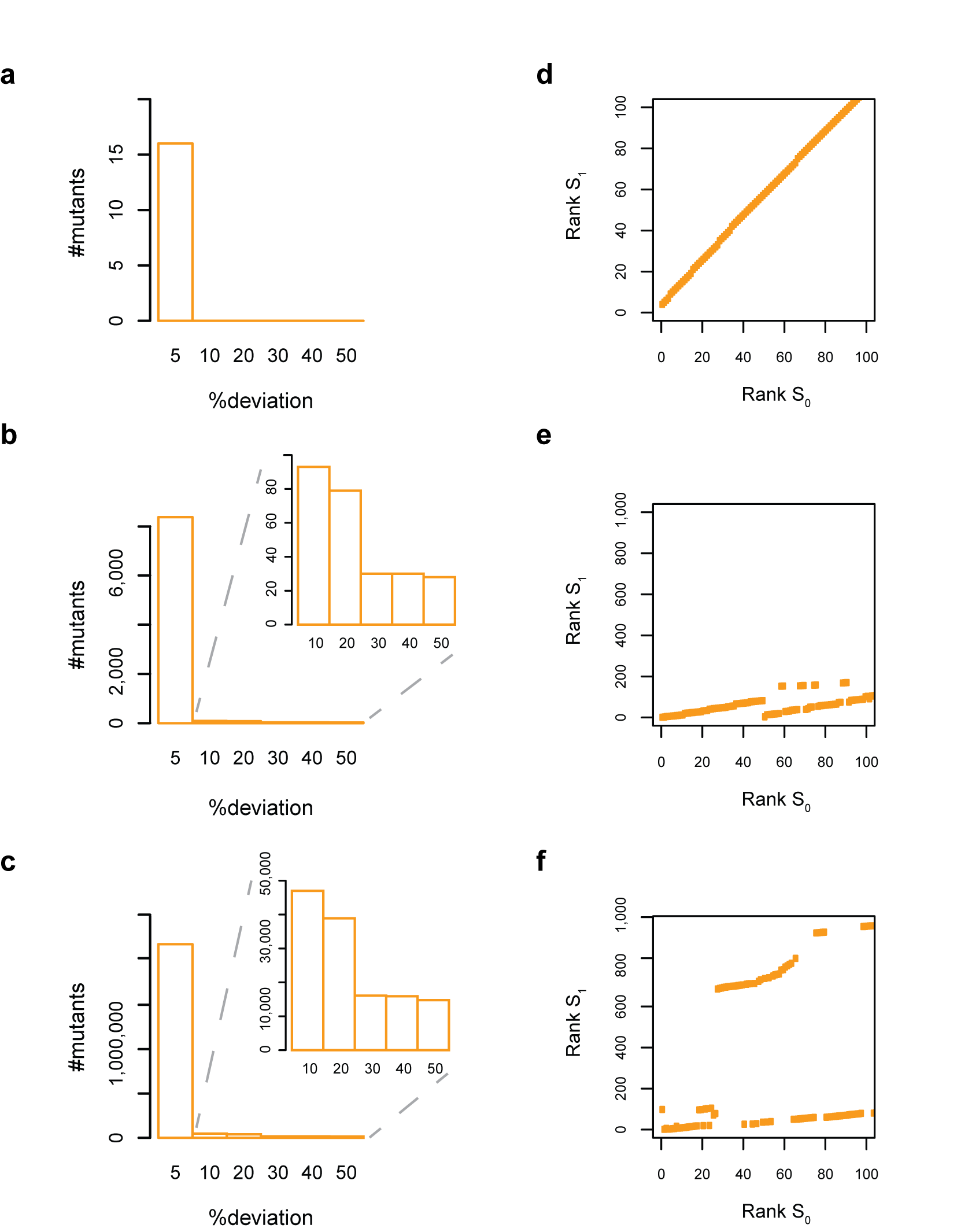

Supplement: Figure S6 — Stoichiometry representation impacts the design of metabolic engineering strategies for improving vanillin-glucoside production in S. cerevisiae depending on the nature of the objective function formulation. Shown is the comparison of predicted vanillin-glucoside yield for a) single, b) double and c) triple gene knockout mutants obtained with two alternative stoichiometric matrices (S0 and S1, Methods). The number of mutants diverging in their lMoMA-predicted vanillin-glucoside yield for the two alternative representations of stoichiometry is represented on the y-axis, while the percentage of deviation of product formation by the mutants relative to S0 is represented on the x-axis. d–f) Comparison of ranks of lMoMA-predicted metabolic engineering strategies for improving vanillin-glucoside production obtained by using S0 and S1 for d) single, e) double and f) triple gene knockout mutants. (TIF) [file pcbi.1002758.s006.tif]

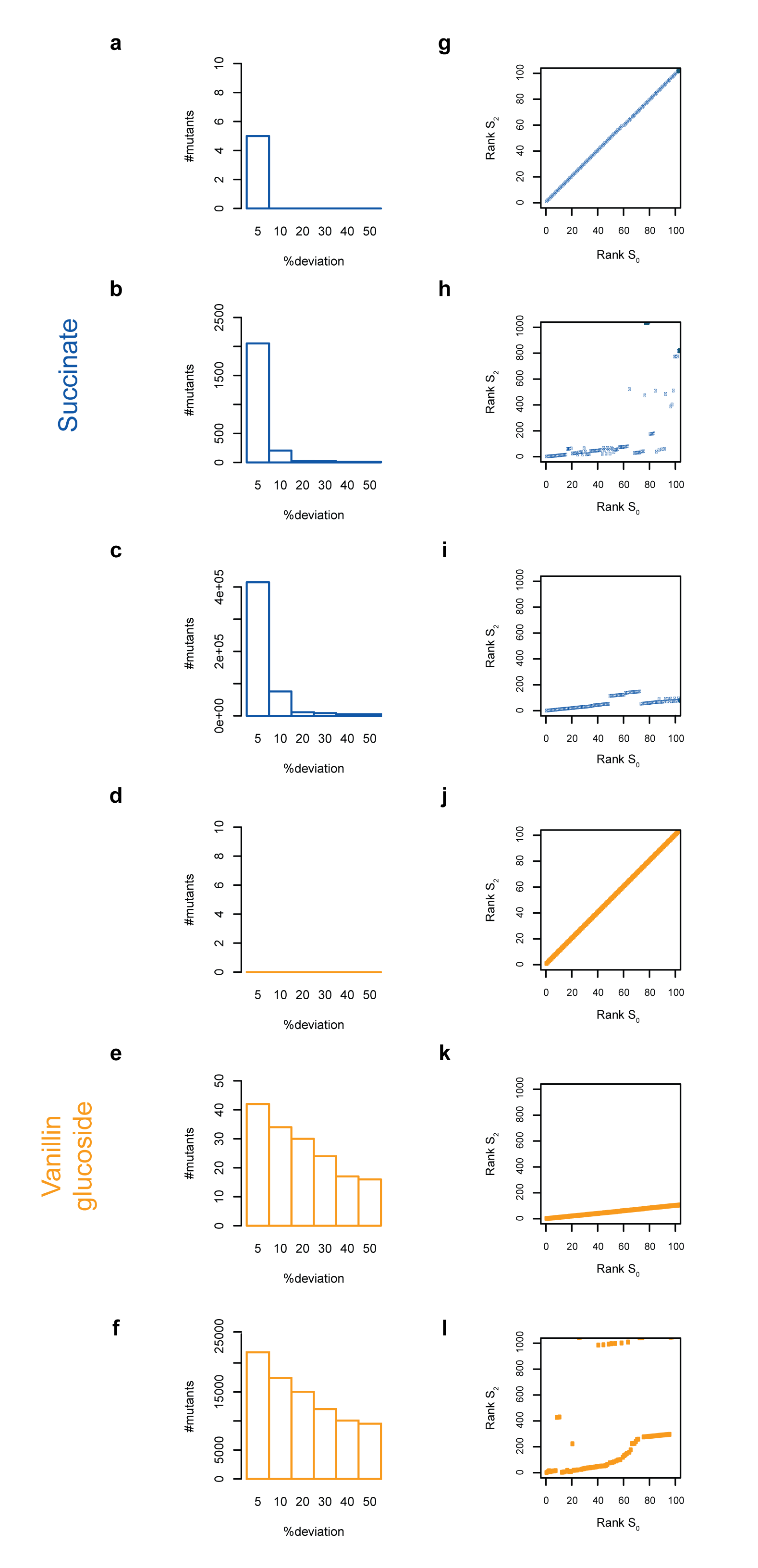

Supplement: Figure S7 — Stoichiometry representation impacts the design of metabolic engineering strategies for improving succinate and vanillin-glucoside yields in S. cerevisiae depending on the nature of the objective function formulation. a–f) Number of mutants diverging in their lMoMA-predicted a–c) succinate and d–f) vanillin-glucoside yields for two alternative representations of stoichiometry, S0 and S2 (Methods). Results for a,d) single, b,e) double and c,f) triple gene knockout mutants are presented. g–l) Comparison of ranks of lMoMA-predicted metabolic engineering strategies for improving g–i) succinate and j–l) vanillin-glucoside production obtained by using S0 and S2. Results for g,j) single, h,k) double and i,l) triple gene knockout mutants are presented. (TIF) [file pcbi.1002758.s007.tif]

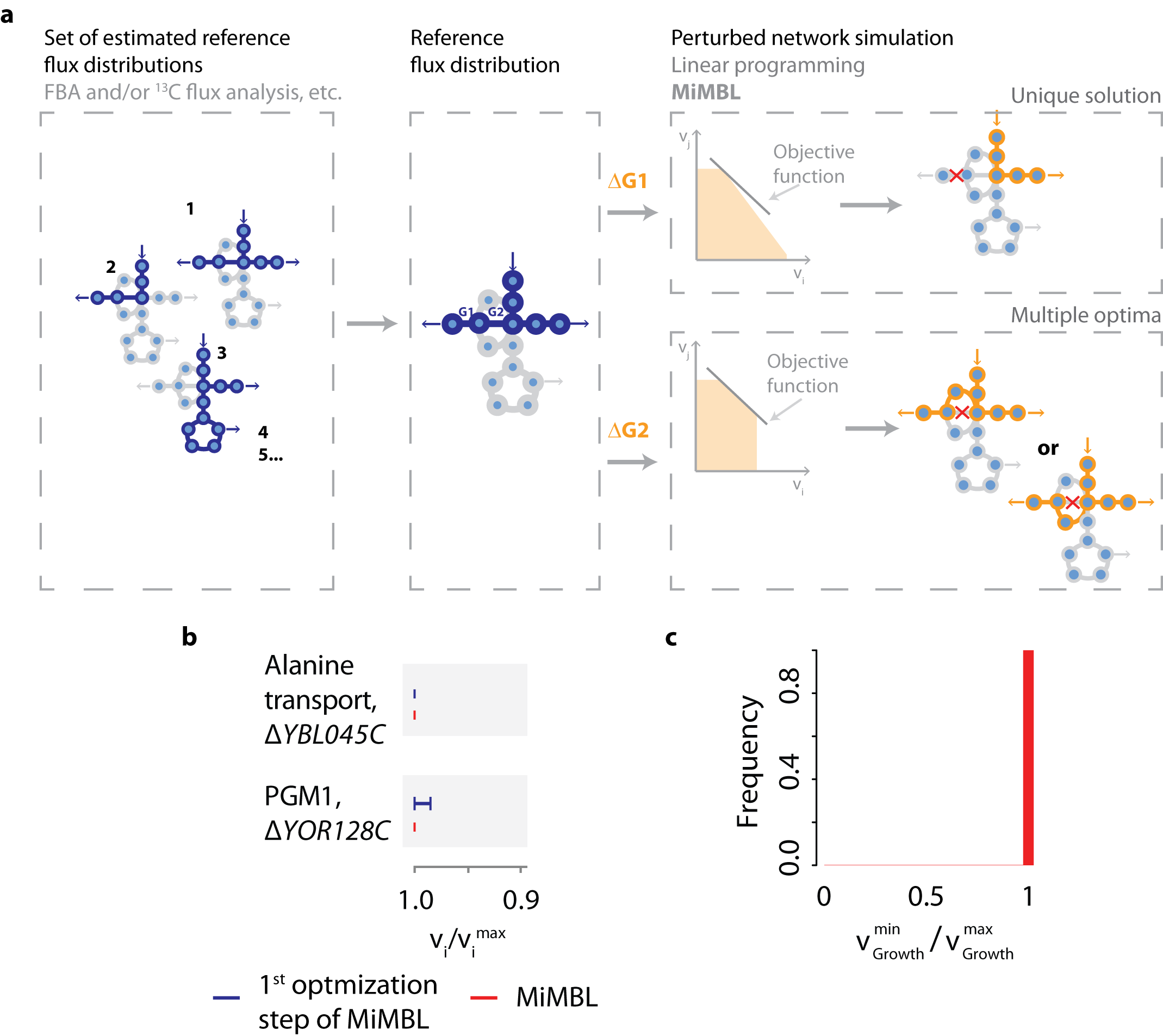

Supplement: Figure S8 — Alternative optima and sensitivity to reference flux distribution. a) The left side of the panel illustrates the variability due to possible uncertainty in the reference flux distribution, for example, as obtained by FBA simulations. The right hand side of the panel illustrates variability in the simulation result owing to the possibility of alternative optimal solutions of the MiMBL linear programming problem. Deletion of Gene 1 illustrates a case where a unique optimal solution is found, while deletion of Gene 2 depicts a case of alternative optima. b) Flux variability analysis to assess the existence of the alternative optimal solutions for a given reference flux distribution (Methods). Shown are the flux variability ranges of alanine transport and flux through phosphoglucomutase (PGM1) after deletion of YBL045C and YOR128C, respectively. PGM1 represents a case where the 2nd optimization step of MiMBL contributes to reducing of flux variability. The corresponding results for lMoMA are presented in Fig. S9. c) Flux variability analysis for growth flux following single/double gene deletions. MiMBL yielded unique growth prediction for single and double gene deletion mutants. Only double gene deletions relevant for the genetic interactions case study (main text) were simulated. (TIF) [file pcbi.1002758.s008.tif]

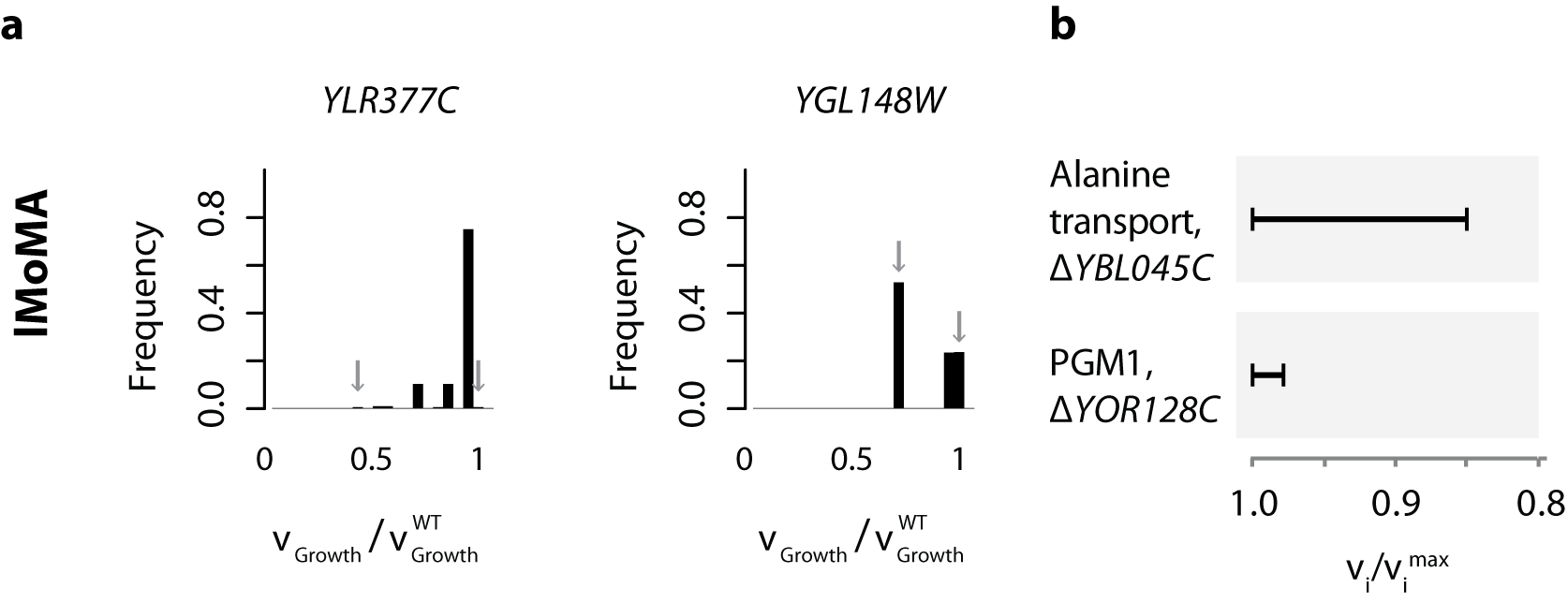

Supplement: Figure S9 — Alternative optima and sensitivity to reference flux distribution: lMoMA. a) Sensitivity of MiMBL towards the use of different reference flux distributions (Methods). Shown are histograms of the simulated growth (vGrowth/) of the mutants lacking YLR377C or YGL148W obtained with MiMBL across 500 simulations using alternatively optimal FBA solutions. Gray arrows mark the minimum and the maximum ratio. b) Flux variability analysis to assess alternative optimal solutions for a given reference flux distribution (Methods). Shown are the flux variability ranges of alanine transport and flux through phosphoglucomutase (PGM1) after deletion of YBL045C and YOR128C, respectively. PGM1 represents a case where the 2nd optimization step of MiMBL contributes to reducing of flux variability. (TIF) [file pcbi.1002758.s009.tif]

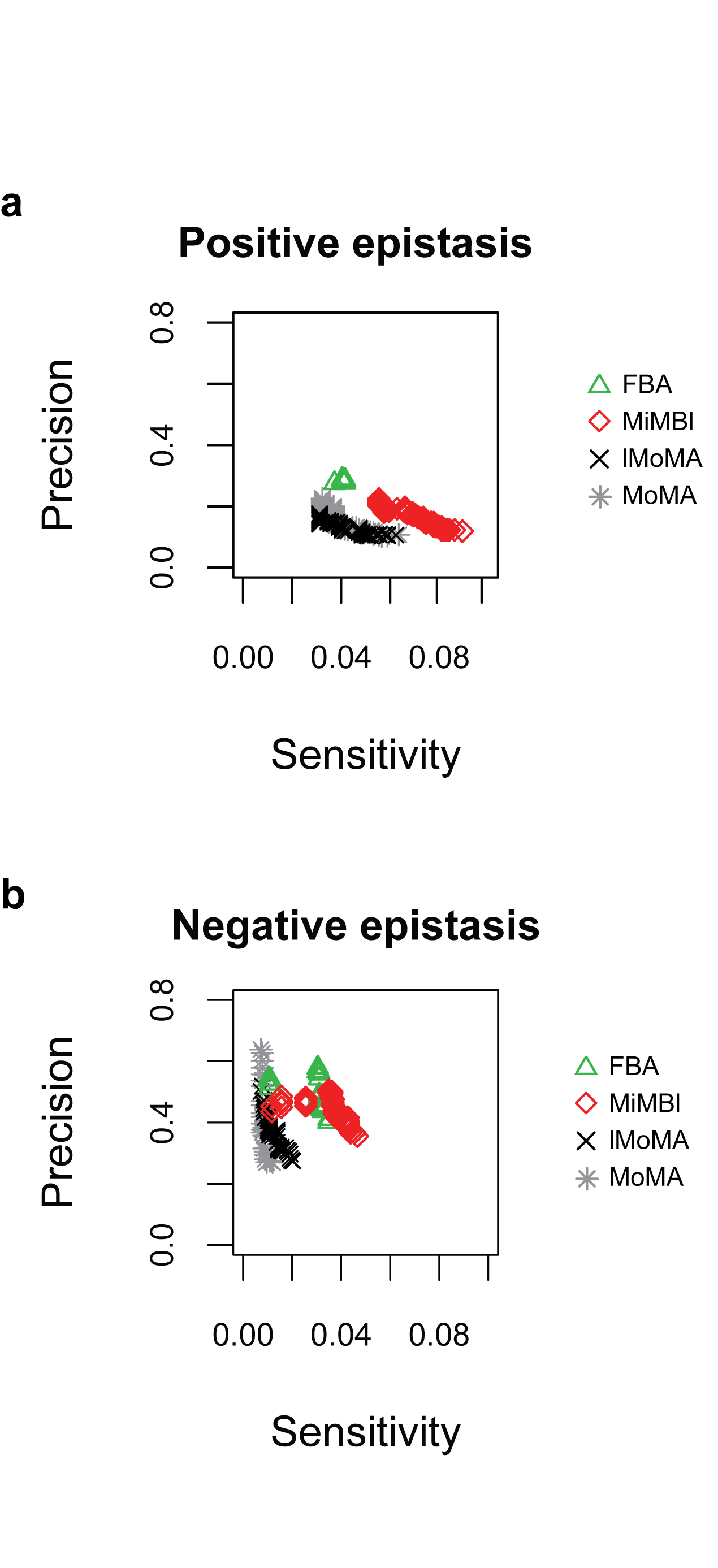

Supplement: Figure S10 — ROC (partial receiver operating characteristic) curves obtained for predicting genetic interactions with MoMA. The ROC curves for the remaining algorithms were kept for reference. Sensitivity reflects the fraction of experimentally validated interactions captured by the algorithm while precision is experimentally validated interactions among all predicted interactions. a) Positive interactions. b) Negative interactions. (TIF) [file pcbi.1002758.s010.tif]

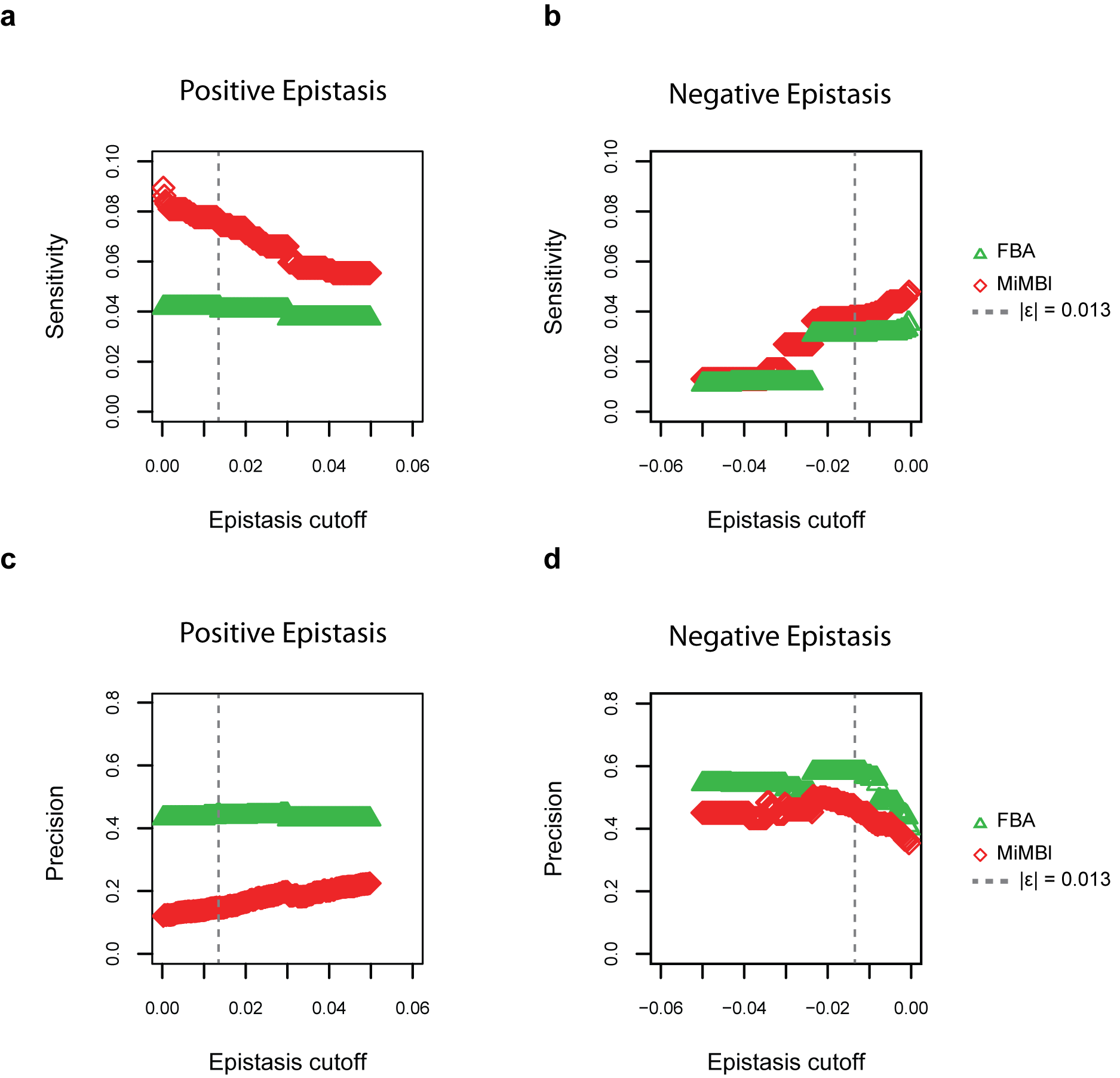

Supplement: Figure S11 — Sensitivity and precision for predicted genetic interactions versus epistasis score cutoff for FBA and MiMBL. The top plots present the sensitivity for positive (a) and negative (b) interactions for FBA and MiMBL. The epistasis score cutoff of |0.13| is represented by a dashed line. The bottom plots present the precision for positive (c) and negative (d) interactions for FBA and MiMBL. The epistasis score cutoff of |0.13| is represented by a dashed line. (TIF) [file pcbi.1002758.s011.tif]
