# Supplementary material for: Impact of Stoichiometry Representation on Simulation of Genotype-Phenotype Relationships in Metabolic Networks
Source: PLoS Comput Biol. 2012 Nov 1;8(11):e1002758. doi: 10.1371/journal.pcbi.1002758 (PMC3486866; doi:10.1371/journal.pcbi.1002758)
Supplement: Table S1 — Number of lMoMA-predicted lethal gene/reaction knockouts in S. cerevisiae that differ between alternative representations of stoichiometry (S1 and S2), relative to S0. (DOCX) [file pcbi.1002758.s012.docx]

**Table S1:** Number of lMoMA-predicted lethal gene/reaction knockouts in *S. cerevisiae* that differ between alternative representation of stoichiometry (*S_1_* and *S_2_*), relative to *S_0_* (**Methods**). The yeast genome scale model [1] was constrained as per Szappanos *et al*, 2011, and FBA was used to generate the reference flux distributions. Single, double and triple gene/reaction deletions were simulated.

| Number of Deletions | ***S_2_*** | | ***S_1_*** | | Total number of combinations | |
| --- | --- | --- | --- | --- | --- | --- |
|  | Reactions | Genes | Reactions | Genes | Reactions | Genes |
| 1 | 0 | 0 | 4 | 3 | 387 | 424 |
| 2 | 0 | 13 | 1526 | 1166 | 74691 | 89676 |
| 3 | 189 | 4565 | 289664 | 225548 | 9585345 | 12614424 |
