# Supplementary material for: Impact of Stoichiometry Representation on Simulation of Genotype-Phenotype Relationships in Metabolic Networks
Source: PLoS Comput Biol. 2012 Nov 1;8(11):e1002758. doi: 10.1371/journal.pcbi.1002758 (PMC3486866; doi:10.1371/journal.pcbi.1002758)
Supplement: Table S2 — lMoMA-predicted epistatic interactions within S. cerevisiae genome-scale metabolic model. (DOCX) [file pcbi.1002758.s013.docx]

**Table S2:** lMoMA-predicted epistatic interactions within *S. cerevisiae* genome-scale metabolic model [1]. Simulations were performed using three alternative representations of stoichiometry, *S_0_*, *S_1_* and *S_2_* (**Methods**). The yeast genome-scale metabolic model was constrained as in Szappanos *et al.* 2011.

|  | ***S_0_*** | ***S_2_*** | ***S_1_*** |
| --- | --- | --- | --- |
| Positive interactions | 2219 | 2154 | 2087 |
| Negative interactions | 840 | 781 | 742 |
| Synthetic lethals | 197 | 217 | 97 |
| Total number of interactions | 89676 | | |
