# Supplementary material for: Impact of Stoichiometry Representation on Simulation of Genotype-Phenotype Relationships in Metabolic Networks
Source: PLoS Comput Biol. 2012 Nov 1;8(11):e1002758. doi: 10.1371/journal.pcbi.1002758 (PMC3486866; doi:10.1371/journal.pcbi.1002758)
Supplement: Text S1 — Supplementary methods. i) Yeast genome-scale metabolic models and simulation conditions; ii) Flux Balance Analysis; iii) Minimization of overall intracellular flux; iv) Minimization of metabolic adjustment – lMoMA; v) Genetic interactions – epistasis score; vi) Metabolic network distance. (DOCX) [file pcbi.1002758.s016.docx]

# Supplementary Methods

All the simulation algorithms were formulated as Linear Programming (LP) optimization problems and implemented in C++ using the GLPK (<http://www.gnu.org/s/glpk/>). Quadratic programming was implemented using IBM ILOG CPLEX Optimizer. All simulations were performed on IBM BladeCenters running CentOS Linux (64bit).

## **Supplementary Method 1:** Yeast genome-scale metabolic models and simulation conditions

The *Saccharomyces cerevisiae* genome-scale stoichiometric reconstruction *i*FF708 [1] was used to study the impact of scaling the stoichiometric matrix on the simulation results when using sum of fluxes in the objective function (**Figs. 1, S4-S7**). The metabolic network was pre-processed so as to remove reactions that carry no flux under the simulated conditions (*i.e.* blocked reactions). Isogenes – genes coding for isoenzymes – were retained. Genes/reactions deemed to be essential by FBA were not considered for knockout simulations within this study, since the prediction of their knockout does not alter when using other algorithms or alternative stoichiometric matrices. Flux Balance Analysis [2] was used to simulate wild-type flux distribution when required, together with the constraints based on experimental data.

For analyzing the predictions of the intracellular flux distribution (**Fig. 1**), *i*FF708 was constrained with physiological data (**including growth**) for a wild-type strain grown in batch cultivation under aerobic conditions [3] and simulations were performed using minimization of overall intracellular flux. In the case studies for determining the impact of alternative stoichiometry representation on the model predictions for growth, genetic interactions and succinate production (**Figs. S4, S5 and S7**), the network was constrained in agreement with the experimental conditions as described by Szappanos *et al.* [4]. In the vanillin case study (**Figs. S4, S6 and S7**), the vanillin-glucoside heterologous pathway according to Brochado *et al.* [5] was introduced in the metabolic network and physiological data from the same study was used to obtain the necessary constraints. The target selection for metabolic engineering was done by simulating the deletion of all possible combinations (up to 3 genes) of (FBA-) non-essential genes. Following the simulations, the metabolic engineering strategies were ranked based on yield on carbon source (glucose) of the product of interest (vanillin or succinate).

The *S. cerevisiae* model *i*AZ900 [6] was used to accomplish the sensitivity analysis towards reference flux distribution, alterative optima analysis (**Fig. 3**) and genetic interactions study (**Fig. 4**). Genes coding for blocked reactions as well as false essentials predicted from single gene deletion simulations were removed from the genetic interactions study, thereby reducing the errors due to misprediction of single-mutant fitness. The metabolic network was constrained in agreement with the experimental conditions as described by Szappanos *et al.* [4].

**Supplementary Method 2:** Flux Balance Analysis [2] was formulated as follows:

$$\begin{matrix} \text{max} v_{Growth} \\ s.t. S\cdot v=0 \\ v_{i}^{lb}\leq v_{i}\leq v_{i}^{ub} \forall i\in N \end{matrix}$$

**Supplementary Method 3:** Minimization of overall intracellular flux [7] was formulated as follows:

$$\begin{matrix} \text{min} \sum_{i} \left|v_{i}\right| \forall i\in N \\ s.t. S\cdot v=0 \\ v_{i}^{lb}\leq v_{i}\leq v_{i}^{ub} \forall i\in N \end{matrix}$$

**Supplementary Method 4:** Minimization of metabolic adjustment (lMoMA, Becker *et al*, 2007) was formulated as follows:

$$\begin{matrix} \text{min} \sum_{i} \left|v_{i}^{WT}-v_{i}\right| \forall i\in N \\ s.t. S\cdot v=0 \\ v_{i}^{lb}\leq v_{i}\leq v_{i}^{ub} \forall i\in N \end{matrix}$$

## **Supplementary Method 5:** Genetic interactions – epistasis score

The fitness (*f*) of each single and double mutant was calculated by normalization of the mutant growth to the wild-type growth. A variety of phenotypic traits can be used to quantify epistasis, growth being the most commonly used, due to its accurate experimental quantification in an efficient high-throughput manner. The genetic interaction score (ε) of each double gene combination (A and B) was calculated based on the following metrics:

$$\varepsilon=f_{AB}-f_{A}\cdot f_{B}$$

To address the accuracy of the different algorithms (FBA, lMoMA and MiMBL) in predicting genetic interactions, we did a *precision versus sensitivity* analysis given the range of computational epistasis score cutoffs |ε_cutoff_|<0.05. *Precision* is the fraction of experimentally validated interactions among all predicted interactions, while the *sensitivity* represents the fraction of the experimentally validated interactions captured by the analysis.

$$Precision= \frac{True positives}{All predicted positives}$$

$$Sensitivity= \frac{True positives}{All experimental positives}$$

All significant genetic interactions among the non-essential genes from Szappanos *et al.* [4] dataset involving genes contained in *i*AZ900 model were included in the present analysis (**Table S4**). The experimental data was filtered by using a confidence threshold of |ε|<0.08 and P<0.05 [4,9].

R 2.12.1 was used to perform statistics calculations and to generate the plots. The Venn diagram from **Fig. 4c** was generated by using the R package “VennDiagram” by Chen & Boutros (2011). Cytoscape 2.8.2 was used to generate the genetic interactions map from **Fig. 4f**.

**Supplementary Method 6:** Metabolic network distance

The metabolic connectivity graph obtained from the metabolic model *i*AZ900 was used to calculate the metabolic/network distance between two genes. We define network distance between the two as the number of reactions belonging to the shortest path between the two genes on the connectivity graph. A pair of directly connected metabolic genes was considered as being separated by distance of 2. This way, genes coding for the same reaction (e.g. isoenzymes and complexes) have a distance of 1. As highly connected metabolites, as cofactors (ATP, NADH, NADPH, FADH2, pyrophosphate and orthophosphate) are not likely to connect genes with related metabolic functions, this subset of metabolites was excluded from the connectivity graph [11]. Despite being highly connected, mitochondrial protons were kept part of the connectivity graph to ensure the integrity of the respiratory chain. Graph Template Library (GTL) was used to implement the algorithm for network distance calculations.

# References

1. Förster J, Famili I, Fu P, Palsson BØ, Nielsen J (2003) Genome-scale reconstruction of the Saccharomyces cerevisiae metabolic network. Genome Research 13: 244–253. doi:10.1101/gr.234503.

2. Varma A, Palsson BØ (1993) Metabolic capabilities of Escherichia coli: I. Synthesis of biosynthetic precursors and cofactors. Journal of Theoretical Biology 165: 477–502.

3. Van Hoek P, Van Dijken JP, Pronk JT (1998) Effect of Specific Growth Rate on Fermentative Capacity of Baker’s Yeast. Applied and Environmental Microbiology 64: 4226–4233.

4. Szappanos B, Kovács K, Szamecz B, Honti F, Costanzo M, et al. (2011) An integrated approach to characterize genetic interaction networks in yeast metabolism. Nature Genetics 43: 656–662. doi:10.1038/ng.846.

5. Brochado AR, Matos C, Møller BL, Hansen J, Mortensen UH, et al. (2010) Improved vanillin production in baker’s yeast through in silico design. Microbial Cell Factories 9: 84. doi:10.1186/1475-2859-9-84.

6. Zomorrodi AR, Maranas CD (2010) Improving the iMM904 S. cerevisiae metabolic model using essentiality and synthetic lethality data. BMC Systems Biology 4: 178. doi:10.1186/1752-0509-4-178.

7. Blank LM, Kuepfer L, Sauer U (2005) Large-scale 13C-flux analysis reveals mechanistic principles of metabolic network robustness to null mutations in yeast. Genome Biology 6: R49. doi:10.1186/gb-2005-6-6-r49.

8. Becker S a, Feist AM, Mo ML, Hannum G, Palsson BØ, et al. (2007) Quantitative prediction of cellular metabolism with constraint-based models: the COBRA Toolbox. Nature Protocols 2: 727–738. doi:10.1038/nprot.2007.99.

9. Costanzo M, Baryshnikova A, Bellay J, Kim Y, Spear ED, et al. (2010) The genetic landscape of a cell. Science 327: 425–431. doi:10.1126/science.1180823.

10. Chen H, Boutros PC (2011) VennDiagram: a package for the generation of highly-customizable Venn and Euler diagrams in R. BMC Bioinformatics 12: 35. doi:10.1186/1471-2105-12-35.

11. Kharchenko P, Church GM, Vitkup D (2005) Expression dynamics of a cellular metabolic network. Molecular Systems Biology 1. doi:10.1038/msb4100023.
