# Supplementary material for: Impact of Stoichiometry Representation on Simulation of Genotype-Phenotype Relationships in Metabolic Networks
Source: PLoS Comput Biol. 2012 Nov 1;8(11):e1002758. doi: 10.1371/journal.pcbi.1002758 (PMC3486866; doi:10.1371/journal.pcbi.1002758)
Supplement: Text S2 — Supplementary notes. i) Toy-model; ii) normlMoMA; iii) Impact of scaling stoichiometry on finding the optimal solution for metabolic flux distributions using FBA-like objective functions – Analytical evidence. (DOCX) [file pcbi.1002758.s017.docx]

# Supplementary notes

**Supplementary note 1: Toy-model**

*Reaction ID Reaction [lower bound, upper bound]*

R1: Axt -> A [1,1]

R2: A -> B [0,100]

R3: B -> C [0,100]

R4: C -> D [0,100]

R5: A -> D [0,100]

R6: A -> D [0,100]

R7: D -> Dxt [0,100]

Where *R1* to *R7* represent reactions, *A, B C* and *D* represent internal metabolites, Axt and Dxt represent external metabolites. Constraints are represented as [*lower bound, upper bound*].

*Reference flux distribution*

R1 1

R2 0

R3 0

R4 0

R5 1

R6 0

R7 1

*Reference intracellular metabolite turnovers*

A 1

B 0

C 0

D 1

**Supplementary note 2: normlMoMA**

One possible approach to normalize the objective function variables with respect to the stoichiometric representation, can be achieved by dividing each variable by its value on the wild-type flux distribution – normalized lMoMA (normlMoMA). Albeit being simple, this normalization method has several drawbacks, the first one being the fact that many reactions have null fluxes on the wild-type flux distribution. The simplest solution to this problem would be to disregard the contribution of null fluxes to the objective function, biologically meaning that the onset of new enzymes would be costless. This normalization solves the inconsistency of the initial problem, since the calculated distance is constant independently of the matrix representation. However, as *R2-R3-R4* and *R6* (toy-model, **Fig. S3**) have null fluxes on the wild-type flux distribution, and therefore they do not take part of the objective function, they represent alternative optima to the problem. Moreover, exclusion of the wild-type null fluxes from the objective function alters the biological principle underlying MoMA and increases the number of alternative optimal solutions.

**Supplementary note 3: Impact of scaling stoichiometry on finding the optimal solution for metabolic flux distributions using FBA-like objective functions – Analytical evidence**

Consider the problem

$$\begin{matrix} \min c_{a}v_{a} \\ s.t. S\cdot v=b \\ v_{i}\geq0, \forall i\in N \end{matrix}$$

Where $a$ is the index of variable $v_{a}$on the matrix $S$. Note that this is a particular case of having a sum of non-normalized variables on the objective function, where all the entries of the objective function coefficients vector $c$ are zero, except for the *a^th^* entry$(=c_{a})$. As described in **Methods**, after scaling matrix $S$with$\Theta$, the optimality condition becomes:

$$c_{j}-c_{B}'\left(B\Theta_{B}\right)^{-1}\theta_{jj}S_{j}\geq0$$

where $j$ is the index of variable $v$ in matrix$S$, $\overline{c_{j}}$is the reduced cost of the variable $v_{j}$, $c_{j}$is the objective function coefficient of$v_{j}$, $c_{B}$is the vector containing the objective coefficients of basic variables, $S_{j}$is the *j^th^* column of matrix $S$, $\Theta$is a $nxn$positive diagonal matrix (**scaling matrix**) and $\theta_{jj}$is the scaling factor for the *j^th^* column of matrix $S$[1].

Three different cases should be considered and in all of them we will show that the **optimality condition is satisfied**.

$$a\notin B and a\neq j$$

The optimality condition is satisfied.

$$a\notin B and a=j$$

$$\begin{matrix} \therefore c_{B}=\left[0\right] \Rightarrow\overline{c_{j}}=c_{j} and c_{j}\geq0 \left(optimality\right) \\ \therefore\overline{c_{j}}_{\Theta}=c_{j}\Rightarrow\overline{c_{j}}_{\Theta}\geq0 \end{matrix}$$

The optimality condition is satisfied.

$$a\in B$$

$$if a=j,then j\in B\Rightarrow\overline{c_{j}}=\overline{c_{j}}_{\Theta}=0$$

$$\begin{matrix} if a\neq j\Rightarrow c_{j}=0 \\ \therefore-c_{B}'\left(B\Theta_{B}\right)^{-1}\theta_{jj}S_{j}\geq0 \end{matrix}$$

As$c_{B}'B^{-1}S_{j}\leq0$and all entries of the matrix $\Theta$are positive, the optimality condition is always satisfied.

# References

1. Bertsimas D, Tsitsiklis JN (1997) Introduction to Linear Optimization. First Edit. Belmont, Massachusetts: Athena Scientific. p.
